# An SI3-σ arch stabilizes cyanobacteria transcription initiation complex

**DOI:** 10.1073/pnas.2219290120

**Published:** 2023-04-10

**Authors:** Liqiang Shen, Giorgio Lai, Linlin You, Jing Shi, Xiaoxian Wu, Maria Puiu, Zhanxi Gu, Yu Feng, Yulia Yuzenkova, Yu Zhang

**Affiliations:** ^a^Key Laboratory of Synthetic Biology, Chinese Academy of Sciences Center for Excellence in Molecular Plant Sciences, Shanghai Institute of Plant Physiology and Ecology, Chinese Academy of Sciences, Shanghai 200032, China; ^b^University of Chinese Academy of Sciences, Beijing 100049, China; ^c^Centre for Bacterial Cell Biology, Biosciences Institute, Faculty of Medical Sciences, Newcastle University, Newcastle upon Tyne NE2 4AX, UK; ^d^Department of Biophysics, Zhejiang University School of Medicine, Hangzhou 310058, China; ^e^Department of Infectious Disease of Sir Run Run Shaw Hospital, Zhejiang University School of Medicine, Hangzhou 310058, China

**Keywords:** gene transcription, cyanobacteria, RNA polymerase, transcription initiation, transcription initiation factor

## Abstract

Multisubunit RNA polymerases (RNAPs) associate with initiation factors (σ in bacteria) to start transcription. The σ factors are responsible for recognizing and unwinding promoter DNA in all bacterial RNAPs. Here, we report two cryo-EM structures of cyanobacterial transcription initiation complexes at near-atomic resolutions. The structures show that cyanobacterial RNAP forms an “SI3-σ” arch interaction between domain 2 of σ^A^ (σ_2_) and sequence insertion 3 (SI3) in the mobile catalytic domain Trigger Loop (TL). The “SI3-σ” arch facilitates transcription initiation from promoters of different classes through sealing the main cleft and thereby stabilizing the RNAP-promoter DNA open complex. Disruption of the “SI3-σ” arch disturbs cyanobacteria growth and stress response. Our study reports the structure of cyanobacterial RNAP and a unique mechanism for its transcription initiation. Our data suggest functional plasticity of SI3 and provide the foundation for further research into cyanobacterial and chloroplast transcription.

## Significance

Catalysis by multi-subunit RNA polymerases (RNAPs) requires correct folding of the flexible Trigger Loop (TL) domain of the active site. The sequence insertion 3 (SI3) in the TL, found in all gram-negative bacteria, is thought to affect multiple stages of transcription via TL folding. Here, we discovered an intrinsic, TL-independent function of SI3. The largest by far SI3 (~65 kDa) of cyanobacteria stretches across the “body” of RNAP and interacts with the initiation factor σ, thus sealing the main cleft of the enzyme. The “SI3-σ” arch formation represents a unique, physiologically relevant mechanism of promoter complex stabilization. This structure-based work in cyanobacteria will advance research into transcription mechanisms in their evolutionary descendants, chloroplasts, which have retained the cyanobacterial machinery.

Most bacterial RNA polymerase core enzymes are composed of five subunits, including two identical α subunits, one β subunit, one β′ subunit, and one  ω subunit (1). Certain gram-positive bacteria can also contain additional accessory δ and ε subunits (2). The bacterial RNAP core enzyme associates with σ factors to form the RNAP-σ holoenzymes that are capable of initiating gene transcription (3). The domain 2 (σ_2_) and domain 4 (σ_4_) of σ^70^-type σ factors are anchored on the β′ coiled-coil motif and the β flap-tip helix of RNAP, respectively, while domain 3.2 (σ_3.2_) is threaded through the active-site cleft of RNAP to connect σ_2_ and σ_4_ (4–6). In the bacterial RNAP holoenzymes, two structural modules—β′ clamp/σ_2_ and β protrusion/lobe—function as pincers to guide, load, and restrain DNA in the main cleft of RNAP. During transcription initiation, σ_2_ nucleates the unwinding process of promoter DNA at the −10 element and recognizes the nucleotide sequence of the unwound −10 element by capturing the −11 and −7 nucleotides of the nontemplate strand in respective protein pockets (7–9).

**Fig. 1.**
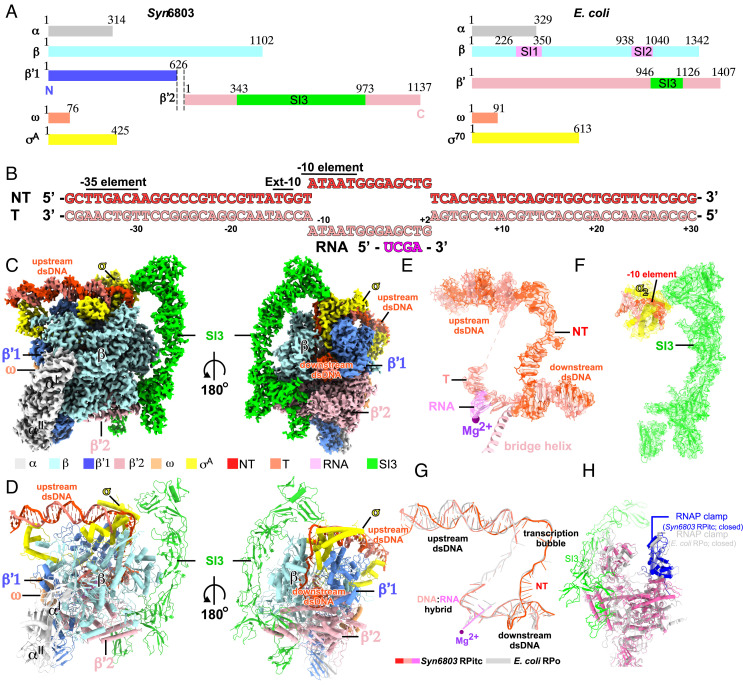
The cryo-EM structure of *Syn*6803 RPitc. (*A*) RNAP subunits of cyanobacteria *Synechocystis* sp. PCC 6803 (*Left*) and *E. coli* (*Right*). (*B*) The nucleic-acid scaffold used in the cryo-EM structure determination. (*C*) Front and back view orientations of *Syn*6803 RPitc cryo-EM map. (*D*) The front and back view orientations of *Syn*6803 RPitc structure. (*E*) The cryo-EM map and model of the nucleic-acid scaffold of *Syn*6803 RPitc. (*F*) The cryo-EM map and model of SI3, σ_2_, and the nontemplate -10 element DNA of *Syn*6803 RPitc. (*G*) Superimposition of the nucleic-acid scaffolds of *Syn*6803 RPitc and *E. coli* RPo. (*H*) Superimposition of the clamp domains of *Syn*6803 RPitc and *E. coli* RPo. RNAP subunits and nucleic-acid chains are colored as in the color scheme.

The composition, working mechanism, and basic transcription factors of bacterial RNAP are conserved across most bacterial species; however, the transcription apparatus is unique in cyanobacterium, an ancient, large, and diverse bacteria phylum. Cyanobacterial RNAP has two distinctive features in comparison to other bacterial RNA polymerases (RNAPs). First, in contrast to a typical bacterial RNAP containing five subunits (2αββ′ω), the *rpoC* gene encoding the full-length RNAP-β′ subunit is split into two genes in cyanobacteria — *rpoC1* and *rpoC2*, resulting in a six-subunit cyanobacterial RNAP (2αβγβ′ω) (10, 11). The *rpoC1* gene encodes the N-terminal half of the full-length RNAP-β′ subunit (γ subunit; referred as β′1 subunit) and the *rpoC2* gene encodes the C-terminal half of the full-length β′ subunit (β′ subunit; referred as β′2) (Fig. 1*A* and *SI Appendix*, Fig. S1) (11). Second, cyanobacterial RNAP contains by far the largest sequence insertion 3 (SI3) “insertion” in its β′2 subunit (630 residues in *Synechocystis* sp. PCC 6803; shortened as *Syn*6803 herein) out of all bacterial RNAPs (10, 12). The 630 residues are inserted between the two helices of the Trigger Loop (TL), the key mobile element in the catalytic center of RNAP that is essential for the catalysis of phosphodiester-bond formation, NTP discrimination, pausing, and cleavage of backtracked RNA (13–18). The TL oscillates between unfolded (inactive) loop and folded Trigger Helix (TH) (catalytically active) conformations*. Escherichia coli* RNAP SI3 (188 residues) has been demonstrated to affect multiple events in transcription initiation, transcription pausing, and intrinsic termination through regulating TL folding (19–23). Compared with *E. coli* RNAP SI3, which comprises two sandwich-barrel-hybrid motifs (SBHMs), the cyanobacterial RNAP-SI3 domain comprises nine SBHMs (12), only one of which shares sequence similarity with *E. coli* RNAP SI3. Notably, chloroplast-encoded RNAP (plastid-encoded RNAP, PEP) retains SI3. Algae PEP SI3 is similar in size to cyanobacterial SI3, and PEP SI3 of higher plants is ~160 residue larger. To date, only the SI3 structure of *E. coli* RNAP has been reported. The structure and function of the largest SI3 insertion of cyanobacterial RNAP remain unknown.

Uniquely for free-living bacteria with complex metabolism, cyanobacteria have a reduced repertoire of basic transcription factors. First, cyanobacterial genomes do not encode any secondary channel-binding factors (10), most of which play crucial roles in *E. coli*: for example, the transcription initiation factor, DksA together with (p)ppGpp, represses ribosomal RNA transcription during nutrient limitation stress (22, 24), and the transcription elongation factors GreA/B facilitate cleavage of RNA in backtracked elongation to restart stalled RNAPs and thus prevent gene expression traffic jams and detrimental collisions with the replication fork (25). We have shown that the intrinsic high hydrolytic activity of cyanobacterial RNA polymerase compensates to a large extent for the absence of these transcription proofreading factors in a recent study (26). Second, cyanobacterial genomes do not encode the termination factor Rho, and thereby gene transcription in cyanobacteria is terminated through the intrinsic termination mechanism. Rho terminates normal and pervasive transcription in *E. coli* (27, 28), and removal of Rho results in severe growth defect in various bacteria (29, 30). It is unknown how cyanobacteria efficiently regulate transcription termination events without Rho.

To understand the molecular mechanism of gene transcription regulation in cyanobacteria, we explored the structural basis of the unique transcription apparatus of cyanobacterial RNAP. We determined the cryo-EM structures of *Synechocystis* sp. Pasteur Culture collection of Cyanobacteria (PCC) 6803 transcription initiation complex (*Syn*6803 RPitc) at a resolution of 3.1 Å and of CTP-bound RPitc at a resolution of 3.0 Å. The structures show that the large SI3 domain extends from the bottom of the secondary channel to the top of the main cleft of RNAP and makes extensive interaction with the rim helices and lobe domain of the RNAP core enzyme and σ factor. Notably, the SI3-head module forms a SI3-σ arch that seals the main cleft of RNAP and stabilizes the transcription initiation complex. Biochemical and genetic evidence suggests the importance of this SI3-σ arch interaction in RNAP-promoter open complex (RPo) formation. Our study provides a structural basis for understanding the intrinsic properties of cyanobacterial RNAP and a foundation for further exploration of gene transcription regulation in cyanobacteria and chloroplasts.

## Results

### The Cryo-EM Structure of Cyanobacterial RPitc.

To obtain the recombinant *Syn*6803 RNAP, we initially coexpressed the six *Syn*6803 RNAP subunits (2α, β, β′1, β′2, and ω subunits) in *E. coli* cells, but failed in obtaining sufficient amounts of recombinant RNAP core enzyme due to poor solubility of the three largest subunits (RNAP-β, β′1, and β′2 subunits). We suspect that the split *Syn*6803 RNAP β′1 and β′2 subunits might be difficult to assemble with other recombinant subunits in *E. coli* cells; therefore, we connected *Syn*6803 RNAP-β′1 and -β′2 subunits with a six-residue flexible linker and were able to obtain a functional recombinant *Syn*6803 RNAP in *E. coli* cells for cryo-EM study (*SI Appendix*, Fig. S2*A* and C).

The *Syn*6803 RPitc complex was reconstituted using the recombinant *Syn*6803 RNAP, *Syn*6803 σ^A^, a nucleic-acid scaffold composed of a 26-bp upstream dsDNA, a premelted 13-bp transcription bubble, a 28-bp downstream dsDNA, and a 4-nt RNA primer (Fig. 1 *A* and *B* and *SI Appendix*, Figs. S1*C* and S2*B*). The structure of *Syn*6803 RPitc was determined at a resolution of 3.1 Å through a cryo-EM single-particle method (*SI Appendix*, Fig. S3). The cryo-EM map exhibits clear signals for all subunits of RNAP and the four major domains (σ_2_, σ_3.1_, σ_3.2_, and σ_4_) of σ^A^ (Fig. 1 *C* and *D*). The cryo-EM map also reveals clear and sharp signals for most nucleotides of the upstream (−37 to −13) and downstream (+3 to +30) dsDNA, all nucleotides (−11 to +2) of the single-stranded nontemplate DNA of the transcription bubble, eight nucleotides (−6 to +2) of the single-stranded template DNA of the transcription bubble, and a 4-nt RNA primer base-paired with template DNA in a post-translocation state (Fig. 1*E *). Our structure shows that in cyanobacterial RPitc, the RNAP-σ^A^ holoenzyme adopts the closed conformation of its clamp domain, induces near 90° bend of the promoter DNA at both junctions of the transcription bubble (Fig. 1 *G* and *H*), and accommodates the promoter DNA as other bacterial RNAP-σ^A^ holoenzymes (Fig. 1*G*) (9).

Our structure shows that the RNAP-β′1 and RNAP-β′2 subunits are split at a loop region located at the surface of RNAP (*SI Appendix*, Fig. S2 *D*–*G*). Both the split ends of the RNAP-β′ subunit (the C terminus of RNAP-β′1 subunit and N terminus of RNAP-β′2 subunit) are well resolved in the cryo-EM map, while the extraneous six-residue linker is disordered, suggesting that the linker does not perturb the local structure’s folds (*SI Appendix*, Fig. S2*F*). Structure superimposition of *Syn*6803 RNAP with other bacterial RNAP reveals essentially the same structural fold and conformation of the two helices at the split ends (*SI Appendix*, Fig. S2*G*). Protein sequence alignment suggests that very few residues are deleted or inserted at the C terminus and N terminus of the RNAP-β′1 and -β′2 subunits, respectively, in various species of cyanobacteria, even though respective genes encoding the two subunits are separated by ~0.5 Mbp in certain species (*SI Appendix*, Fig. S1). Altogether, our structure shows that the two largest subunits (β′1 and β′2 subunits) of *Syn*6803 RNAP exhibit structural fold and interaction with the rest of RNAP at the split point that are similar to those of the unsplit RNAP-β′ subunit.

### Cyanobacterial RNAP-SI3 Encloses a Large RNAP Surface.

The cryo-EM map reveals a strong signal for the *Syn*6803 RNAP-SI3 domain, but local resolutions of most regions span from 4.5 to 7.0 Å except for the subregions making contacts with the rest of the RNAP holoenzyme (Fig. 1*F* and *SI Appendix*, Fig. S3*F*). Since the low resolution of the RNAP-SI3 domain does not permit ab initio model building, we determined a 1.6 Å crystal structure of the SI3-tail domain (residues 352 to 433; corresponding to *Syn*6803 residues 343 to 424) of *Synechococcus elongatus* PCC 7942 (*Syn*7942) RNAP that shares 45% protein sequence identity with the SI3-tail domain of *Syn*6803 RNAP (*SI Appendix*, Fig. S4*A* and Table S1). Furthermore, we took advantage of the crystal structure of *Thermosynechococcus elongatus BP-1* RNAP SI3 (residues 435 to 983; corresponding to *Syn*6803 residues 430 to 977; PDB: 8EMB) and built a near-complete structure model of SI3 domain into the cryo-EM map of *Syn*6803 RPitc.

**Fig. 2.**
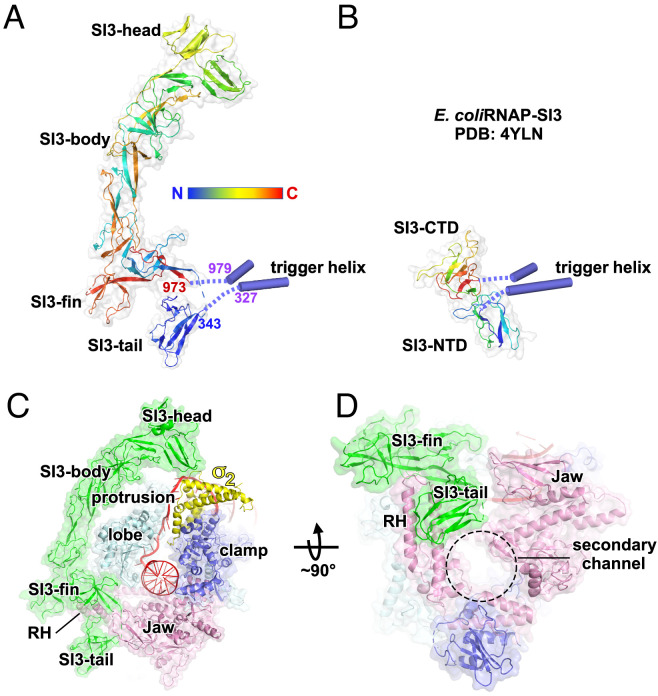
The structure of *Syn*6803 RNAP-SI3. (*A*) The overall structure of *Syn*6803 RNAP-SI3. The Trigger Helices connect to the SI3-tail and SI3-fin domains. (*B*) The overall structure of *E. coli* RNAP-SI3 (PDB: 4YLN). (*C*) SI3 encloses half of the RNAP surface. It extends from the secondary channel to the top of main cleft and makes interaction with σ_2_. (*D*) The SI3-fin and SI3-tail domains shield RNAP Rim Helices.

Cyanobacterial RNAP SI3 folds into a seahorse-shaped structure that can be further divided into four domains: SI3-tail (residues 343 to 424), SI3-fin (residues 884 to 973 and 424 to 478), SI3-body (residues 478 to 619 and 729 to 884), and SI3-head (residues 619 to 729) (Fig. 2*A*). The *Syn*6803 RNAP SI3-body and SI3-head domains are absent in *E. coli* RNAP SI3, while the SI3-tail and SI3-fin domains resemble the two domains of *E. coli* RNAP SI3 (SI3-NTD and SI3-CTD) that are located near the secondary channel (Fig. 2*B*). The SI3-tail domain of *Syn*6803 RNAP adopts a structural fold similar to SI3-NTD of *E. coli* RNAP [(rmsd 1.7 Å of 296 Cα atoms; *SI Appendix*, Fig. S4*D*)], while the SI3-fin domain of *Syn*6803 RNAP adopts a structural fold radically different from SI3-CTD of *E. coli* RNAP (rmsd 3.6 Å of 364 Cα atoms). In contrast to the two subdomains of *E. coli* RNAP SI3 which stably associate with each other, the SI3-tail and SI3-fin domains of *Syn*6803 RNAP SI3 barely contact each other, which presumably permits independent movement of SI3-tail (Fig. 2 *A* and *B*).

The SI3 domain of *Syn*6803 RNAP contacts the rest of the RNAP holoenzyme through multiple surface patches. The SI3-tail and SI3-fin bind the loop and stem of the rim helices, respectively; SI3-fin contacts the β-lobe loop that stabilizes the downstream dsDNA; SI3-body extends to the top of the main cleft and shields the lobe and protrusion domains; and SI3-head interacts with σ^A^ (Fig. 2 *C* and *D*).

### Cyanobacterial RNAP-SI3 Forms an SI3-σ Arch with σ^A^.

**Fig. 3.**
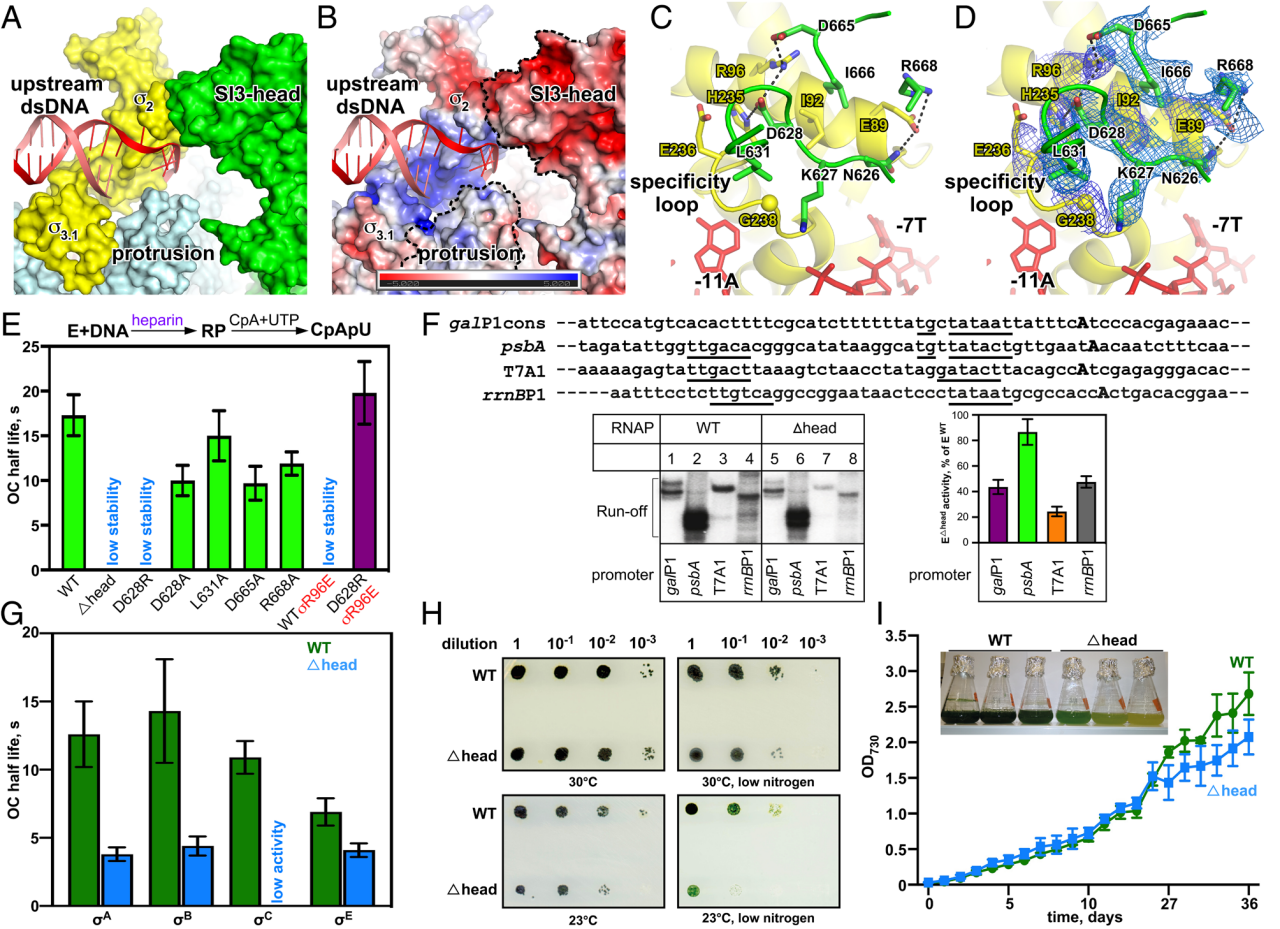
The structural and functional analysis of SI3-σ arch. (*A*) Surface presentation of the SI3-σ arch. (*B*) Electrostatic surface presentation of the SI3-σ arch. (*C*) Detailed interaction between SI3 and σ_2_. (*D*) The cryo-EM map of the SI3-σ_2_ interface. (*E*) The half-lives of RNAP-promoter open complex comprising *gal*P1cons promoter and indicated holoenzymes challenged with heparin. Error bars represent SD from triplicate experiments. “Low stability” indicates that decay rates were too high to measure accurately. (*F*) Activity of E^WT^ and E^Δ^^head^ on different promoters. *Top*, promoter sequences with transcription start site shown in bold and consensus elements underlined; *Bottom*, representative gel (*Left*) and bar plot (*Right*) show amounts of runoff transcripts of E^Δ^^head^ in % from that of E^WT^. Error bars represent SD from three independent experiments. (*G*) The half-lives of RNAP-promoter open complexes comprising WT/Δhead RNAP and different σ factors. Error bars represent SD from triplicate experiments. (*H*) Serial dilutions of cultures of *S. elongatus* 7942 WT strain and strain with genomic deletion of SI3 (Δhead) plated on BG-11 media and grown at conditions indicated below images with constant light. (*I*) Growth curves of WT and Δhead *S. elongatus* 7942 strains in 12 h light/12 h dark conditions. Error bars represent SD from three independently grown cultures. The *Inset* is an image of flasks after 33-d growth.

The SI3-head domain of RNAP tethers σ^A^ by making interactions with the σ^A^_1.2_ helix and the specificity loop of σ^A^_2_, the key structural motifs that recognize the flipped-out −11 nucleotide within the −10 element of promoter DNA to initiate dsDNA melting. The SI3-σ interaction forms an arch-like structure that inhibits opening of the RNAP clamp and provides a physical obstacle that prevents the single-stranded DNA of the transcription bubble from dissociating and rewinding (Fig. 3*A*). Moreover, the negatively charged surface of the SI3-σ arch helps restrain the promoter DNA by electrostatic repulsion in the positively charged groove underneath (Fig. 3*B*). Although *Syn*6803 RNAP SI3 does not contact the promoter DNA, it fills in a shallow cavity between the specificity loop and σ^A^_1.2_ helix, likely stabilizing the active conformation of the specificity loop for accommodating the flipped −11A nucleotide of the nontemplate strand of promoter DNA (Fig. 3 *C* and *D*).

Detailed interactions of the SI3-σ interface include two salt bridge bonds made by D628 and D665 of the SI3-head and R96 of σ^A^, one salt bridge bond made by R668 of SI3 and E89 of σ^A^, one H-bond made by N626 of the SI3-head and E89 of σ^A^, and one H-bond made by D628 of the SI3-head and H235 of σ^A^. Moreover, residues K627, L631, and I666 of SI3 and residues I92, E236, and G238 of σ^A^ contribute to the interactions through Van der Waals forces (Fig. 3 *C* and *D*). The interface residues are conserved across different cyanobacteria species, suggesting physiological relevance of the SI3-σ interaction (*SI Appendix*, Fig. S5 *A* and *B*).

Contacts seen in the cryo-EM structure between the SI3-head and σ^A^_2_ are expected to stabilize RPo. To test this hypothesis, we challenged transcription on the *gal*P1cons promoter with the DNA competitor heparin. We used wild type (WT) *Syn*7942 RNAP holoenzyme along with mutants bearing either the SI3 head domain deletion (E^Δ^^head^) or single amino acid substitution of the SI3 residues that make contacts with σ in the structure – E^D628A^, E^D628R^, E^L631A^, E^D665A^ and E^R668A^. The *gal* P1cons promoter, a model promoter of the extended –10 type that is predominant in cyanobacteria (31), was chosen to test the mutants. The RPo complexes were challenged with heparin (10 μg/mL) for increasing time intervals prior to the addition of substrates for synthesis of the 3-nt RNA transcripts (Fig. 3*E* and *SI Appendix*, Fig. S6*A*). The RPo half-life was calculated from the decay plots of the activity (*SI Appendix*, Fig. S6*A*). Transcription reports directly on promoter complex half-life in this experiment, since the steady-state rate of synthesis of the short abortive transcript is proportional to the amount of RPo complexes, and 10 μg/mL concentration of heparin does not affect catalysis of nucleotide addition. E^Δhead^, E^D628R^ and E^σR96E^ showed a substantial decrease in RPo half-life, and the actual decay rates were too high to measure accurately (Fig. 3*E*). Notably, the impaired RPo stability of E^D628R^ was fully restored to WT level in the E^D628R/σR96E ^holoenzyme, in which a reciprocal point amino acid change in σ, R96E was introduced to restore the salt bridge bond (Fig. 3*E*). Control experiments showed that the mutant RNAP core enzyme (E^Δhead^) exhibited the same catalytic rate and affinity to σ as those of the WT RNAP core enzyme (*SI Appendix*, Fig. S7). Overall, these results validate the interactions seen in the cryo-EM structure and highlight the importance of SI3-σ arch interaction on RPo stability.

The above results show that the SI3-σ arch increases RPo stability. To investigate the effect of SI3-σ arch on overall transcription activity, we measured the activity of the WT and E^Δhead^ RNAP holoenzymes with representative promoters, including chloroplast P_   *psbA* (a strong promoter with near-consensus −35, extended −10, and discriminator elements and an optimal 17-bp −35/−10 spacer), *E. coli* P_  *gal* P1cons promoter (extended −10 promoter), bacteriophage T7 promoter P_T7A1 (−35/−10 promoter), and *E. coli* ribosomal P_*rrn*B P1 (−35/−10 promoter) (Fig. 3*F*). The results showed that disruption of the SI3-σ arch interaction significantly decreased the activity of RNAP on the P_T7A1, P_*rrn*B P1, and P_   *gal* P1cons promoters but had little effect on P_  *psb*A (Fig. 3*F*). The results suggested that the SI3-σ arch interaction might be crucial for maintaining transcription activity of weak promoters and has less impact on strong promoters (i.e., P_  *psbA*).

Sequence alignment of σ^A^ with other alternative σ factors shows that the σ^A^ residues, which make interactions with SI3, are conserved in four σ^70^-type group-II σ factors in *Syn*6803 (σ^B^, σ^C^, σ^D^ and σ^E^), but not in three group-IV σ factors in *Syn*6803 (σ^G^, σ^H^ and σ^I^) and the group-III σ factor (σ^F^), suggesting that SI3 likely also makes interactions with the four alternative group-II σ factors (*SI Appendix*, Fig. S5*A*). Supporting this prediction, the stability of RNAP-promoter DNA complex comprising E^Δhead^ and σ^70^-type group II-σ factors σ^B^, σ^C^ and σ^E^ (*rpoD*2, 4, and 6 of *Syn*7942) was impaired to different extents compared with that of the respective WT RNAP holoenzymes (Fig. 3*G*). The stability of the open complex is lower for the holoenzymes formed with σ^E^ even for E^WT^, consistent with changes of both Glu89 and Arg96 residues participating in the formation of SI3-σ arch to Gln residues in this σ (*SI Appendix*, Fig. S5*A*). Intriguingly, the SI3-σ interface does not appear to be conserved in plant chloroplast RNAP (*SI Appendix*, Fig. S5*B*). As the SI3 domains of chloroplast RNAPs are larger in many plant species compared with those of cyanobacteria, detailed structural information is required to see whether the SI3-σ arch is preserved in chloroplast RNAP.

To investigate the physiological role of the SI3-σ arch, we constructed a chromosomal deletion of the SI3 head domain in *Syn*7942 and tested the growth phenotype of the resulting strain. Under optimal laboratory growth conditions, there was no obvious growth defect of this mutant on solid media at constant light (Fig. 3*H*). However, compromised growth of the SI3 head mutant strain was observed under stress-inducing conditions, such as low temperature, nitrogen deprivation, or their combination (Fig. 3*H*). In liquid culture with light conditions mimicking diurnal rhythms (12-h light followed by 12-h darkness), the WT and mutant strains initially grew at a similar rate, but the mutant strain entered stationary phase prematurely with bleached cultures after 20-d growth (Fig. 3*I*). Altogether, these data suggest that the SI3-σ arch plays an important role in bacterial growth under nutrient-limited conditions, as well as during stress responses.

### Cyanobacterial RNAP-SI3 Interacts with the Rim Helices.

**Fig. 4.**
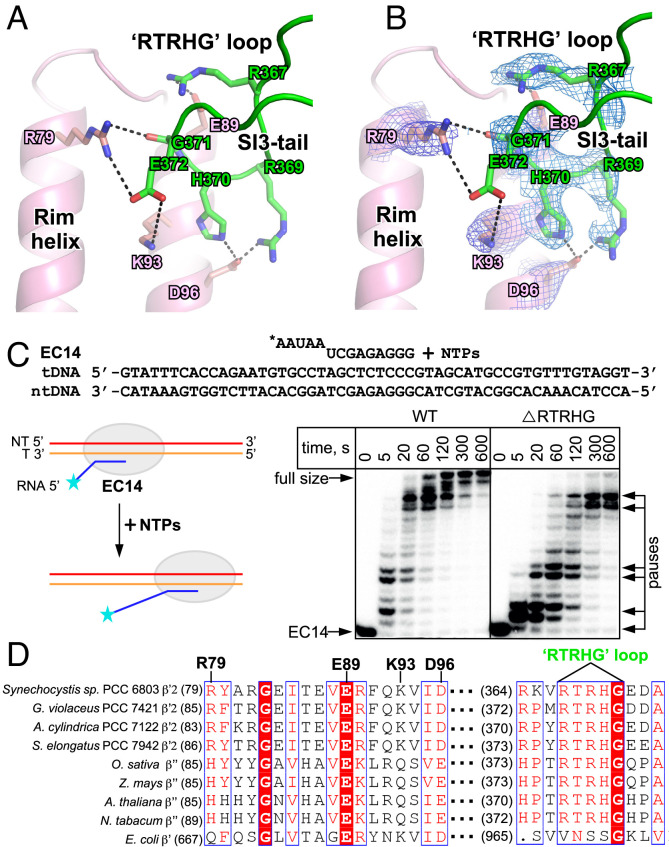
SI3 interacts with the rim helices. (*A*) Detailed interaction between the rim helices and SI3-tail. (*B*) The cryo-EM map of the SI3-rim interface. (*C*) The RTRHG loop deletion leads to increased pausing during elongation. Elongation complex is assembled with 14-nt long RNA labeled at 5′-end with ^32^P and template and nontemplate DNA oligonucleotides fully complementary to each other. NTPs were added to a final concentration of 10 μM, reaction stopped at indicated timepoints with addition of formamide-containing loading buffer. (*D*) The protein sequence alignment of the SI3-tail domain of various cyanobacterial RNAP and plant chloroplast PEPs.

The cryo-EM structure of cyanobacterial RPitc reveals that the SI3-tail makes extensive interactions with the rim helix hairpin near the secondary channel of RNAP (Figs. 2*D* and 4 *A* and *B* ). The SI3-tail is a barrel-sandwich hybrid domain composed entirely of β strands. The “RTRHG” loop (named after the five conserved resides of the loop) protrudes out from the main “body” of the SI3-tail and contacts the stem of the rim helix hairpin (Fig. 4*A*). The conserved residues R367, R369, H370, and G371 of the RTRHG loop make an H-bond network with four principal residues (R79, E89, K93, and N96) of the rim helix (Fig. 4 *A* and *B*). The SI3-rim interface is conserved in all aligned cyanobacterial RNAP and chloroplast PEPs in various plants, but it is not conserved in *E. coli* and other bacterial species (Fig. 4*D* and *SI Appendix*, Fig. S4 *C* and *D*), suggesting a lineage-specific interaction. Disruption of this interaction by deletion of the RTHRG loop led to increased ubiquitous pausing during elongation and an inability for RNAP to reach the end of the template (Fig. 4*C*). This result suggests that disruption of SI3-rim interactions allows direct influence of SI3 movement on TL function in the active site (*Discussion*).

### The Conformational Change of SI3 upon TL Refolding.

**Fig. 5.**
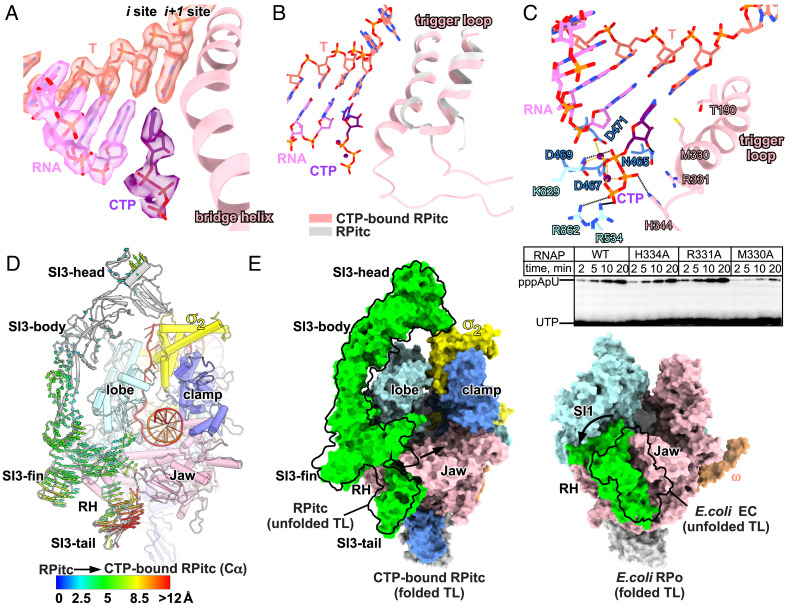
TL refolding induces structural change in *Syn*6803 NTP-bound RPitc. (*A*) The cryo-EM map and model for the active site. CTP adopts an insertion state at the “i+1” site. (*B*) CTP induces refolding of TL into TH. (*C*) The *Top* panel shows the detailed interaction between CTP and RNAP residues; the *Bottom* panel shows the effect of alanine substitutions of CTP-contact residues on the efficiency of the first phosphoric diester bond formation during de novo initiation on *gal*P1cons promoter. (*D*) The conformational changes of SI3 induced by TL refolding (cluster of arrows showing domain movement). (*E*) The comparison of SI3 movement upon TL refolding between *Syn*6803 RNAP (*Left*) and *E. coli* RNAP (*Right*).

The large SI3 insertion is located between the two helices of the TL (Fig. 2*A*), the key structural element that undergoes folding/unfolding during each nucleotide-addition cycle (16, 32). In the *Syn*6803 RPitc structure, the TL is in an unfolded state, probably due to absence of NTP at the “i+1” site. To study whether refolding of the TH affects SI3 conformation and its interaction with RNAP, we sought to determine the structure of *Syn*6803 NTP-bound RPitc. We first reconstituted *Syn*6803 RPitc with a modified RNA primer, where the 3′ terminal nucleotide was replaced by a 3′-deoxyadenosine. We subsequently incubated the *Syn*6803 RPitc with CTP and determined the cryo-EM structure of *Syn*6803 CTP-bound RPitc at 3.0 Å resolution (*SI Appendix*, Fig. S8). The cryo-EM map shows that Cytidine triphosphate (CTP) occupies the “i+1” site (Fig. 5*A*). The α phosphate of CTP is in close distance with the C3′ atom of the “i” site adenine, indicating that it adopts an insertion state ready for incorporation (Fig. 5*A*).

CTP binding induces refolding of the TL into the TH (Fig. 5*B*). The refolded TH forms a salt bridge and H-bond interactions with the phosphate groups of CTP essentially the same as in the crystal structure of *T. thermophilus* Cytidine-5′-[(α,β)-methyleno]triphosphate, a non-hydrolyzable CTP analog (CMPCPP)-bound transcription elongation complex (TEC) (16), except that the invariant histidine (H334), which functions as a positional catalyst (13), contacts the β phosphate in our structure instead of the α phosphate in *T. thermophilus* CMPCPP-bound TEC (Fig. 5*C* and *SI Appendix*, Fig. S9*B*). In agreement with our structure, mutant RNAP with an M330A change has a strong effect on the formation of the first diester bond between the initiating ATP and UTP on the *gal*P1cons promoter, presumably due to loss of its stacking interaction with the base of the incoming nucleotide (Fig. 5*C*). R331A and H334A substitutions have a lesser effect (Fig. 5*C*).

Upon TL refolding, the conformations of the SI3-head and -body domains remain unchanged, but the SI3-fin and -tail domains undergo a rotational movement toward the secondary channel (Fig. 5*D*). The SI3-σ arch remains intact, suggesting that nucleotide addition does not disrupt the SI3-σ arch interaction (*SI Appendix*, Fig. S9*C*). Interaction between the “RTRGH” loop of the SI3-tail and β′ rim helices also remains intact, since the β′ rim helices, SI3-fin, and SI3-tail rotate as a single structural unit (*SI Appendix*, Fig. S9 *D* and *E*). The TH refolding-induced stretching of the two short linkers, L1 and L2, that connect the TH to the SI3-tail and SI3-fin domains likely accounts for this domain rotation (*SI Appendix*, Fig. S9*F*). Compared with the large conformational change of *E. coli* RNAP SI3 upon TH refolding (20, 33), this structural module in cyanobacteria RNAP rotates to a much lesser extent (Fig. 5*E*).

## Discussion

Cyanobacteria are the only prokaryotes capable of oxygenic photosynthesis (34). As a result, they oxygenated the atmosphere of the Earth ~ 2.3 billion y ago, changing the subsequent evolutionary course of the entire biosphere; they gave rise to chloroplasts ~2.1 billion y ago (34, 35). Here, we report the architecture of the cyanobacterial RNAP and pave the road for further understanding and engineering of the transcription apparatus in cyanobacteria. Moreover, since cyanobacterial RNAP is the ancestor of plastid-encoded RNAPs, our work provides foundation to understand the structure and evolution of chloroplast RNAP.

We show that SI3 contacts a conserved surface patch on the σ_2_ domain of the group-I σ factor σ^A^ and the alternative group-II σ factors in cyanobacteria, including σ^B^, σ^C^, σ^D^, and σ^E^ (*SI Appendix*, Fig. S5*A*). The surface patch of σ_1.2_ and σ_2_ interacting with SI3 locates nearby but does not overlap with regions interacting with Crl, RbpA, and GcrA factors (of γ-proteobacteria, actinobacteria, and α-proteobacteria, respectively) on respective σ factors (36–40). We show that disruption of the SI3-σ arch affects transcription of tested holoenzymes containing group-I or -II σ factors, and therefore likely affects transcription initiation of a large proportion of genes. We have shown that the impaired SI3-σ arch causes growth defect of cyanobacteria in nitrogen-depleted conditions. Cyanobacteria do not possess specialized σ^54^ regulating nitrogen metabolism. Therefore, this growth defect could be attributed to the cumulative effect of several σ factors involved, but σ^C^ might play a bigger role than other σ factors as the RNAP-σ^C^ holoenzyme is the one mostly affected upon disruption of the SI3-σ arch. σ^C^ is evolutionary and functionally conserved in cyanobacteria, and it has been reported to control the expression of key nitrogen regulatory genes in several species, including *Syn*6803 and *Syn*7942 (41). Further detailed study is required to globally evaluate the contribution of the SI3-σ arch on cyanobacterial gene expression.

Folding of the TL upon substrate addition is completed without SI3-σ arch disruption or large conformational change of SI3 at the stage of transcription initiation, in contrast to *E. coli* SI3 (20). After RNAP escapes from the promoter and enters the elongation stage, the SI3-σ arch is broken, and SI3 body and “head” may become more mobile and potentially available for interactions with cellular factors. Could these interactions generate a signal for SI3 to transmit onto the TL, and affect catalysis? Perhaps not, at least not uncontrollably. We suggest that the TL is insulated from effects of SI3 conformational changes by SI3 attachment to the main body of RNAP at the base of the NTP entry channel. Disruption of this interaction via RTRHG loop deletion leads to increased pausing in elongation due to the effect of thermal motion of SI3 on TL folding. Moreover, this attachment and the lack of major conformation change in SI3 upon NTP binding implies a low probability of its rhythmic movement with every nucleotide addition cycle, like the proposed movement of the much smaller *E. coli* SI3 (42). At the same time, we cannot exclude that, under specific conditions, the interface between the rim helices and the RTRHG loop could be targeted by regulatory factors to influence catalysis by the TL.

In summary, we present here the structures of cyanobacterial transcription initiation complexes. These structures reveal an unexpected SI3-σ arch interaction that stabilizes RPo and maintains bacterial growth in nutrient-limited environments. Further structures of transcription elongation and termination complexes of cyanobacterial RNAP are required to illustrate the regulatory mechanisms and role of SI3 at these stages of transcription.

## Material and Methods

Detailed descriptions of protein purification, complex assembly, cryo-EM specimen preparation, cryo-EM data acquisition and processing, model building and refinement, in vitro transcription assays, and bacterial growth phenotyping experiments are provided in *SI Appendix*.

## Supplementary Material

Appendix 01 (PDF)

## Data Availability

The cryo-EM maps and coordinates were deposited in Protein Data Bank and Electron Microscopy Data Bank (*Syn*6803 RPitc: 8GZG and EMD-34397; *Syn*6803 CTP-bound RPitc: 8GZH and EMD-34398; *Syn*7942 RNAP SI3-tail: 8H02 (43–47)). All other data are included in the manuscript and/or *SI Appendix*.
