# Supplementary material for: An SI3-σ arch stabilizes cyanobacteria transcription initiation complex
Source: Proc Natl Acad Sci U S A. 2023 Apr 10;120(16):e2219290120. doi: 10.1073/pnas.2219290120 (PMC10120043; doi:10.1073/pnas.2219290120)
Supplement: Supplementary file 1 — Appendix 01 (PDF) [file pnas.2219290120.sapp.pdf]

Supplementary Information for

A SI3-σ arch stabilizes cyanobacteria transcription initiation complex

Liqiang Shen[1,2,#], Giorgio Lai[3,#], Linlin You[1,2,#], Jing Shi[4], Xiaoxian Wu[1], Maria Puiu[3], Zhanxi Gu[1,2], Yu Feng[4,*], Yulia Yuzenkova[3,*], Yu Zhang[1,*]

[*]Correspondence: yzhang@cemps.ac.cn (Y.Z.); Yulia.Yuzenkova@newcastle.ac.uk (Y.Y.); yufengjay@zju.edu.cn (Y.F.)

This PDF file includes:

SI Materials and Methods

Figures S1 to S9

Tables S1 to S4

SI Material and methods

Plasmids

The pET28a-TEV-*rpoD* was constructed by inserting the *Syn*6803 *rpoD* gene into pET28a-TEV plasmid using restriction sites NcoI and NotI. The pET28a-TEV-*Syn*7942 *rpoC2* SI3-tail was constructed by inserting the DNA fragment encoding *Syn*7942 RNAP SI3-tail (residues 350-433) into pET28a-TEV plasmid using restriction sites NcoI and NotI. The pACYCduet-*Syn*6803-*rpoAZ* was constructed by inserting the *Syn*6803 *rpoA, rpoZ* genes into the pACYCDuet (Merk Millipore) using restriction site pairs BamHI/NotI and NdeI/XhoI, respectively. The pETDuet-*Syn*6803-*rpoBC2* was constructed by inserting the *Syn*6803 *rpoB, rpoC2* genes into pETDuet plasmid (Merk Millipore) using restriction site pairs NcoI/NotI and NdeI/XhoI, respectively. The pETDuet-*Syn*6803-*rpoBC1C2*(3xGS) was constructed by inserting the *Syn*6803 *rpoC1* gene into pETDuet-*Syn*6803-*rpoBC2* through homologous recombination, resulting in a fused *rpoC1C2 peptide* by a 3xGS-linker.

Syn7942 genes *rpoA*, *rpoB*, *rpoC1*, *rpoC2*, and *rpoZ* were PCR amplified from genomic DNA. Primers were design so as to add RBSs sequences before each gene and a 7-residues long poly-histidine tag at *rpoC2* C-terminus. After purification, the PCR products were ligated in pJET1.2/blunt (ThermoFischer Scientific) with T4 DNA Ligase (New England Biolabs) according to the manufacturer's instructions. The resulting plasmids were named pJET-A, pJET-B, pJET-C1, pJET-C2, and pJET-Z respectively, and were used as template DNA for downstream PCRs. *RpoB* was PCRed and assembled onto pJET-A by Gibson Assembly to yield pJET-AB, then *rpoC1* was assembled onto pJET-AB to yield pJET-ABC1. *RpoZ* was assembled onto pJET-C2 to yield pJET-C2Z. *RpoC2* and *rpoZ* were then assembled onto pJET-ABC1 to yield pJET-ABC1C2Z. Finally, the operon containing all five genes was PCR amplified from pJET-ABC1C2Z and assembled onto plasmid pET28a to yield pET28a-SelRNAP.

To obtain pET28a-SelRNAP-ΔSI3head, plasmid pET28a-Syn7942 RNAP was PCR amplified with primers flanking the head of β' SI3 domain. The resulting PCR product was re-circularized by Gibson Assembly without addition of any insert. All Gibson Assembly reactions were performed using NEBuilder® HiFi DNA Assembly Master Mix according to the manufacturer's instructions. Briefly, 50 ng of backbone PCR were mixed with a two-fold molar excess of insert and 2.5 µL NEBuilder® HiFi DNA Assembly Master Mix in 5.0 µL final volume. The reaction was incubated at 50°C for 1 hour before transformation in NEB5α cells. Genes expressing His_6-tagged RpoD1, RpoD2, RpoD4, and RpoD6 were cloned separately into pET28 expression vector. Detailed information of the plasmids and primers used in this study are listed in Tables S3 and S4.

*Syn*6803 RNAP

The recombinant *Syn*6803 RNAP core enzyme was purified from *E. coli* BL21(DE3) (Novo protein, Inc.) cells carrying pACYCDuet-*Syn*6803-*rpoAZ* and pETDuet-*Syn*6803-*rpoBC1C2*(3xGS). Protein expression was induced at an OD_{600} of 0.6-0.8 by 1 mM IPTG at 18 ºC for 16 h. Cells were lysed in lysis Buffer A (50 mM Tris-HCl, pH 7.7, 200 mM NaCl, 5% glycerol, 2 mM EDTA, 2 mM DTT, 0.1 mM phenylmethylsulfonyl fluoride (PMSF) and protease inhibitor cocktail (Biomake.cn. Inc.)) using an Avestin EmulsiFlex-C3 cell disrupter (Avestin, Inc.). The supernatant of lysate was precipitated by 0.6% (v/v) polyethylenimine (PEI). The pellet was collected and RNAP was extracted from the pellet with buffer 50 mM Tris-HCl, pH 7.7, 5% glycerol, 1 M NaCl, 2 mM DTT, and 2mM EDTA. The RNAP solution was further precipitated by addition of ammonium sulfate (final concentration; 29 g/mL). The pellet was collected and RNAP was extracted in NTA-binding buffer (20 mM Tris-HCl, pH 7.7, 5% glycerol, 400 mM NaCl, 5 mM β-mercaptoethanol). The supernatant was loaded on to a 5 mL column packed with Ni-NTA agarose (SMART, Inc.). The bound-RNAP was washed with 50 mL NTA-binding buffer containing 20 mM imidazole and eluted with Ni-NTA buffer containing 500 mM imidazole. The eluted fractions were mixed with TGED buffer (20 mM Tris-HCl, pH 7.7, 5% glycerol, 2 mM DTT, 2 mM EDTA) at ratio 1:1 and loaded onto a Mono Q column (MonoQ 10/100 GL, Cytiva) followed by a salt gradient of buffer A (20 mM Tris-HCl, pH 7.7, 200 mM NaCl, 5% (v/v) glycerol, 1 mM DTT) and

buffer B (20 mM Tris-HCl, pH 7.7, 600 mM NaCl, 5% (v/v) glycerol, 1 mM DTT). The fractions containing target proteins were collected, concentrated to 5 mg/mL, and stored at -80 ºC.

***Syn*6803 σ^A**

The recombinant *Syn*6803 σ^A was purified in *E. coli* BL21(DE3) cells carrying pET28a-TEV-*rpoD*. The protein expression was induced with 0.4 mM IPTG at 18 ºC for 16 h when OD_{600} reached 0.6-0.8. Cell pellet was lysed in lysis buffer B (50 mM Tris-HCl, pH 7.7, 500 mM NaCl, 5% (v/v) glycerol, 5 mM β-mercaptoethanol, and 0.1 mM phenylmethylsulfonyl fluoride (PMSF)) using an Avestin EmulsiFlex-C3 cell disrupter. The supernatant was loaded on to a 2 mL column packed with Ni-NTA agarose (Smart-lifesciences, Inc.). The bound proteins were washed by the lysis buffer B containing 20 mM imidazole and eluted with the lysis buffer B containing 400 mM imidazole. The eluted fractions were subjected to TEV protease cleavage while dialyzing to buffer 20 mM Tris-HCl, pH 7.7, 200 mM NaCl, 5% (v/v) glycerol, 5 mM β-mercaptoethanol. The sample was reloaded onto the Ni-NTA column to remove impurity. The sample was diluted, loaded onto a MonoQ column, and eluted with a salt gradient of buffer A (20 mM Tris-HCl, pH 7.7, 0.1 M NaCl, 5% (v/v) glycerol, 1 mM DTT) and buffer B (20 mM Tris-HCl, pH 7.7, 0.5 M NaCl, 5% (v/v) glycerol, 1 mM DTT). The fractions containing target proteins were collected, concentrated to 10 mg/mL, and stored at -80 ºC.

***Syn*7942 RNAP core enzyme and σ subunits.**

RNAP core enzyme and σ subunits were separately expressed in T7express cells (NEB). Briefly, the cultures were grown in LB at 37 °C until OD_{600} ~0.6, transferred to 20 °C, induced with 1 mM IPTG, and further grown for 4 hours. Cells were collected, resuspended in lysis buffer (50 mM Tris HCl pH 8.0, 250 mM NaCl, 10% glycerol, 20 mM imidazole, 1 mM β-mercaptoethanol, and protease inhibitors cocktail (Roche, according to manufacturer's instruction) and lysed by sonication, all steps were done at 4 °C. Soluble fraction was recovered by centrifugation for 15 minutes at 17000 g and applied to 5 ml HiTrap Q HP column (Cytiva). Fractions containing core subunits were eluted with lysis buffer containing 200 mM imidazole. Pooled eluate fractions were loaded onto pre-equilibrated Strep-Tactin XT gravity flow column. Column was washed with buffer (100 mM Tris HCl pH 8.0, 150 mM NaCl, 1 mM EDTA), protein eluted with same buffer containing 2.5 mM d-Desthiobiotin. Fractions containing pure core enzyme (judged by SDS PAGE) were pooled, concentrated using Amicon Ultra 100 kDa cut-off centrifugal device, and dialysed overnight against storage buffer (40 mM Tris HCl pH 8.0, 200 mM KCl, 50% glycerol, 1 mM EDTA, 1 mM DTT). σ subunits were isolated using HiTrap Q HP and Superdex 200 chromatography (Cytiva), fractions were pooled, proteins were concentrated and dialysed against the same storage buffer. Mutant RNAP core enzyme and σ subunits were produced using side-directed mutagenesis and same purification steps as for WT proteins.

***Syn*6803 RNAP-σ^A holoenzyme**

*Syn*6803 RNAP core enzyme and *Syn*6803 σ^A were incubated in a ratio of 1:4 at 4 °C for 4 h. The mixture was applied to a Superdex 200 Increase 10/300 GL column (Cytiva) equilibrated in 20 mM Tris-HCl, pH 7.7, 150 mM NaCl, 1 mM DTT. Fractions containing *Syn*6803 RNAP holoenzyme was collected and concentrated to 10 mg/mL.

***Syn*7942 RNAP SI3-tail**

The *Syn*7942 RNAP SI3-tail were purified in *E. coli* BL21(DE3) cells carrying pET28a-TEV-*Syn*7942-SI3tail by a similar procedure as above except an additional purification step on a HiLoad 16/60 superdex 75 pg column (Cytiva). The protein in 10 mM Tris-HCl, pH 7.7, 50 mM NaCl, 1 mM DTT were collected and concentrated to 40 mg/mL.

Nucleic-acid scaffolds.

The pre-melted promoter used for cryo-EM study of *Syn*6803 RPitc and CTP-bound RPitc contains consensus sequences of the -35, -10, and discriminator elements. The nucleic-acid scaffold was prepared from synthetic oligonucleotides (sequences in Figs. 1A and S8A) by an annealing procedure (95°C, 5 min followed by 2°C-step cooling to 25°C) in annealing buffer (20 mM Tris-HCl, pH 8.0, 200 mM NaCl).

Crystal structure determination of *Syn*7942 RNAP SI3-tail

The initial screen of *Syn*7942 RNAP SI3-tail was performed by a sitting-drop vapor diffusion method. Crystals were grown from drops containing 1 µL 40 mg/mL protein and 1 µL reservoir solution (0.2 M ammonium acetate, 0.1 M Tris pH 8.5, 25% w/v polyethylene glycol 3,350) at 22 °C. Crystals were transferred into the reservoir solution containing 15% (v/v) (2R, 3R)-(−)-2,3-butanediol (Sigma-Aldrich) and flash-cooled in liquid nitrogen. Data were collected at Shanghai Synchrotron Radiation Facility (SSRF) beamline 19U1, processed using HKL2000 (1). The structure was solved by molecular replacement with Phaser MR using the structure of *E. coli* β' SI3 (PDB: 2AUK) as a search model (2). Cycles of iterative model building and refinement were performed in Coot (3) and Phenix (4). The final model of *Syn*7942 RNAP SI3-tail was refined to Rwork and Rfree of 0.210 and 0.233.

Cryo-EM structure determination of *Syn*6803 RPitc

*Syn*6803 RNAP holoenzyme and the nucleic-acid scaffolds were incubated in a ratio of 1:1.5 at room temperature for 15 min. The mixture was applied to a Superdex 200 Increase 10/300 GL column (Cytiva) equilibrated in 20 mM Tris-HCl, pH 7.7, 150 mM NaCl, 1 mM DTT. Fractions containing *Syn*6803 RP**itc** were collected and concentrated to 10 mg/mL. *Syn*6803 RPo was subsequently mixed with CHAPSO (Hampton Research, Inc.) to a final concentration of 8 mM prior to grid preparation. About 3.5 µL sample was applied onto the glow-discharged C-flat CF-1.2/1.3 400 mesh copper grids (Electron Microscopy Sciences) and the grid was handled and plunge-frozen in liquid ethane using a Vitrobot Mark IV (FEI) with 95% chamber humidity at 10 °C.

Data were collected on a 300 keV Titan Krios (FEI) equipped with a K2 Summit direct electron detector (Gatan). A total of 3432 images were recorded using the Serial EM in super-resolution mode with a pixel size of 0.507 Å, and a dose rate of 53.6 electrons/pixel/s. Movies were recorded at 250 ms/frame for 8 s (32 frames total) and defocus range was varied from -1.5 µm to -2.6 µm. Frames in individual movies were aligned using MotionCor2 (5), and contrast-transfer-function estimations were performed using CTFFIND4 (6). About 1,000 particles were picked and subjected to 2D classification in RELION 3.0 (7). The resulting distinct two-dimensional classes were served as templates and a total of 473,353 particles were picked out. The resulting particles were manually inspected and subjected to 2D classification in RELION 3.0 by specifying 100 classes. A 60 Å low-pass-filtered map was calculated from structure of *E. coli* RPo (PDB: 4YLN) as the starting reference model for 3D classification (8). A total number of 158,150 particles were used for constructing the final cryo-EM map. The final maps were further subjected 3D auto-refinement, CTF-refinement, Bayesian polishing, and post-processing in RELION 3.0 (Fig. S3). Gold-standard Fourier-shell-correlation analysis (FSC) indicated a mean map resolution of 3.14 Å. The x-ray structure of *E. coli* RPo (PDB: 4YLN) was manually fit into the cryo-EM density map using Chimera. Model building and real-space refinement were performed in Coot (3) and Phenix(4).

Cryo-EM structure determination of *Syn*6803 CTP-bound RPitc

*Syn*6803 RNAP core enzyme and σ^A were incubated in a ratio of 1:4 at 4 °C temperature for 4 h. The mixture was applied to a Superdex 200 Increase 10/300 GL column (Cytiva) equilibrated in 10 mM Hepes, pH 7.5, 100 mM KCl, 5mM $MgCl_2$, 3 mM DTT. Fractions containing *Syn*6803 RNAP holoenzyme were collected and concentrated to 14 mg/mL. *Syn*6803 RNAP holoenzyme (30 µM) was mixed with the nucleic-acid scaffold (45 µM) with a molar ratio of 1:1.5 for 1 h, and subsequently supplemented with CTP (3 mM) allowing incubation at 4 °C temperature for 4 h. The CTP-bound RPitc was mixed with CHAPSO (Hampton Research, Inc.) to a final concentration 8 mM prior to grid preparation. About 3 µL mixture was applied on a glow-discharged UltraAuFoil R1.2/1.3 300 mesh holey Au grids (Quantifoil Micro Tools GmbH), blotted with Vitrobot Mark IV (FEI), plunge-frozen in liquid ethane with 100% chamber humidity at 22 °C.

The micrographs were collected using EPU in the super-resolution counting mode on a 300 keV Titan Krios (FEI) equipped with a Gatan K3 Summit direct electron detector (pixel size 1.10 Å/pixel). A total of 2,297 images were recorded using the Super-resolution mode (exposure, 2.69 s per 40-frame movie; dose rate, 22.5 electrons/pixel/s; defocus, -1.2 to -2.2 µm). Frames in individual movies were aligned

using MotionCor2 (5), and contrast-transfer-function estimations were performed using CTFFIND4 (6). About 1,000 particles were picked and subjected to 2D classification in RELION 3.0 (7). The resulting distinct two-dimensional classes were served as templates and a total of 681,334 particles were picked out.

The particles were subjected to 2D classification in RELION 3.0 by specifying 100 classes. A 50 Å low-pass-filtered map was calculated from cryo-EM map of *Syn*6803 RPitc as the starting reference model for 3D classification (N=6). One 3D class with distinct shape of RNAP containing 120,655 particles was subjected to 2D classification again for generating templates for the second round of particle auto-picking. A total of 338,818 particles were auto-picked by using the 2D references. The particles were subjected to 2D classification in RELION 3.0 by specifying 100 classes. A total of 264,653 particles were selected and subjected to 3D classification using a 50 Å low-pass-filtered cryo-EM structure of the previous 3D classification (N=4). One 3D class with distinct shape of RNAP containing 145,731 particles were used for constructing the final cryo-EM map. The final maps were further subjected 3D auto-refinement, CTF-refinement, Bayesian polishing, and post-processing in RELION 3.0. Gold-standard Fourier-shell-correlation analysis (FSC) indicated a mean map resolution of 2.96 Å. The structure of *Syn*6803 RPitc was manually fit into the cryo-EM map using Chimera. Model building and real-space refinement were performed in Coot (3) and Phenix(4).

Growth and mutagenesis of *Syn*7942

Wild type and mutant strains of *Syn7942* were grown in BG-11medium, at either constant light or 12-hour light/12-hour dark cycle as indicated in the legend of Figure 3 at 100 µE light intensity at 30 °C unless overwise indicated, in AlgaeTron 130 incubator. Liquid cultures were shaken at 300 rpm. Solid media was prepared using 1.2% gellan gum. Nitrogen starvation was induced by lowering down the concentration of $NaNO_3$ in media to 10 µM.

To generate the mutant *S. elongatus* strains β'ΔSI3head and β'WT, the corresponding editing plasmids pUC-β'ΔSI3head and pUC-β'WT were transformed in wild type *S. elongatus* cells as previously described (9). Briefly, 15 mL of *S. elongatus* culture at OD_{730} 0.6-0.7 were pelleted by centrifugation at 6,000 g for 10 minutes. Cells were then washed once in 10 mL 10 mM NaCl and resuspended in 0.3 mL BG-11 medium. After addition of 0.5-5.0 µg of plasmid, cells were incubated overnight at 30 °C in the dark with gentle shaking and finally seeded on nitrocellulose membranes (purchased from Merck) laid on BG-11 agar plates supplemented with selective antibiotic. The plates were incubated under constant light at 30 °C and the membranes were transferred to fresh BG-11 agar plates every 2-4 days until colonies appeared. Colonies were then re-streaked several times until full segregation of the mutation was achieved (as assessed by PCR).

***In vitro* transcription**

All reactions for *Syn7942* RNAPs were done at 30 °C on linear PCR-derived templates containing promoters indicated on figures, in transcription buffer (20 mM Tris-HCl pH 7.9, 40 mM NaCl, 10 mM $MgCl_2$). Reactions contained 30 nM RNAP core enzyme, 100 nM σ, and 100 nM *gal*P1cons promoter fragment. For testing susceptibility of promoter complexes to heparin treatment, 0.01 mg/mL heparin was added for 20”, 1’, 2’, 5’, and 10’ before substrates addition. Transcription was initiated with 100 µM CpA and 40 µM [α-^{32}P] radiolabeled UTP (7.5 Ci/mmol). Reactions were allowed to proceed for 5 min and then they were terminated by the addition of an equal volume of loading buffer (1X TBE, 8M Urea, 20 mM EDTA, 100 µg/mL heparin, 0.02 % bromophenol blue, 0.02 % xylene cyanole in formamide). Reaction products were resolved by electrophoresis in 23% denaturing polyacrylamide gel, visualized by PhosphorImager (Cytiva), and quantified using the ImageQuant software (Cytiva). Half-life of the promoter complex was calculated by fitting the data into exponential decay equation $f = y_0+a*exp(-b*x)$ using non-linear regression by SigmaPlot software, where x is the reaction time, f is the quantified 3-nt transcripts, y_0, a, and b are unconstrained constants.

For testing the activity of RNAP on different promoters, the run-off transcription reactions contained 250 µM ATP, CTP, GTP and 25 µM [α-^{32}P] UTP (5 Ci/mmol) were incubated for 10 minutes before termination with loading buffer.

Reaction of dinucleotide product formation on *gal*P1cons promoter were performed with 500 µM ATP and 25 µM [α-^{32}P] UTP (5 Ci/mmol), kept for the time intervals indicated in Figure, stopped with formamide-containing loading buffer and resolved in 33 % denaturing polyacrylamide gel.

Transcription elongation kinetics experiments were performed using assembled elongation complexes essentially as described in (10). Briefly, elongation complex is assembled with 14 nt long synthetic oligonucleotide RNA labelled at 5'-end with [γ-^{32}P] ATP and fully complementary template and non-template DNA oligonucleotides, sequences of all shown on Figure 4C. Assembled complexes were immobilized on Ni-NTA Sepharose beads via hexa-histidine tag on RNAP and washed with 1M NaCl-containing transcription buffer to remove any aberrantly assembled complexes. NTPs were added to final concentration of 10 µM, reaction started with addition of 10 mM $MgCl_2$ and stopped at timepoints indicated on Figure 4C with addition of formamide-containing loading buffer. Reaction products were resolved and visualized as before.

Microscale thermophoresis

σ^A was fluorescently labelled on amines with NT647 RED-N-hydroxysuccinimide (NHS) reactive dye (Nanotemper), according to manufacturer's protocol (Nanotemper). A fixed concentration of 400 nM σ^A was used in a set of 16 serial dilutions testing a range of core RNAP concentrations (from 0.5 nM to 5 µM) in MST buffer (40 mM Tris-HCl pH 7.9, 20 mM KCl, 10 mM MgCl2, 5% glycerol). 5 µL of each reaction was loaded into premium capillaries, and MST was performed at 80% fluorescence excitation power on a Monolith NT.115. Binding curves were plotted and K_d estimated using NT Analysis 1.5.41 and Affinity Analysis software.

Supplemental figures

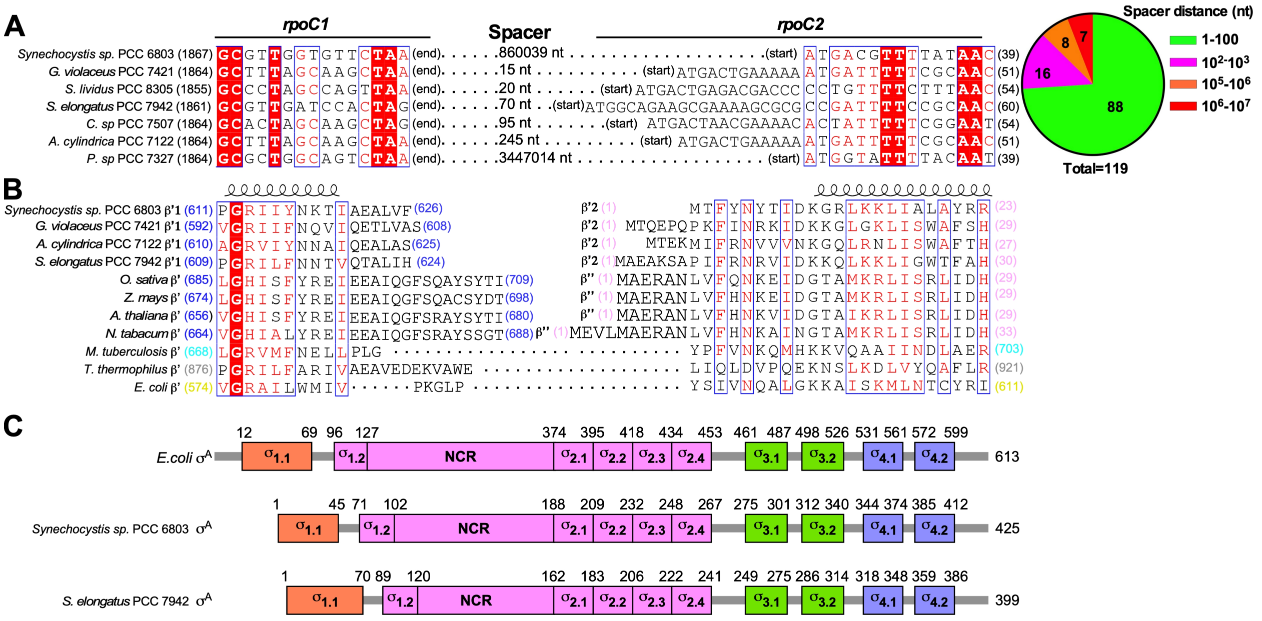

Figure S1. Sequence features of RNAP subunits and σ^A. (A) The DNA sequence alignment of *rpoC1* and *rpoC2* genes from representative cyanobacteria. The pie chart at right shows the spacer distribution of the two genes at 119 non-redundant cyanobacteria genomes. **(B)** The protein sequence alignment of RNAP-β'1 and -β'2 subunits from representative cyanobacteria, plastid-encoded polymerase (PEP) β' and β'' subunits of representative plant chloroplasts, and other eubacteria species. **(C)** The domain features of *E. coli* σ^{70}, *Syn*6803 σ^A, *Syn*7942 σ^A.

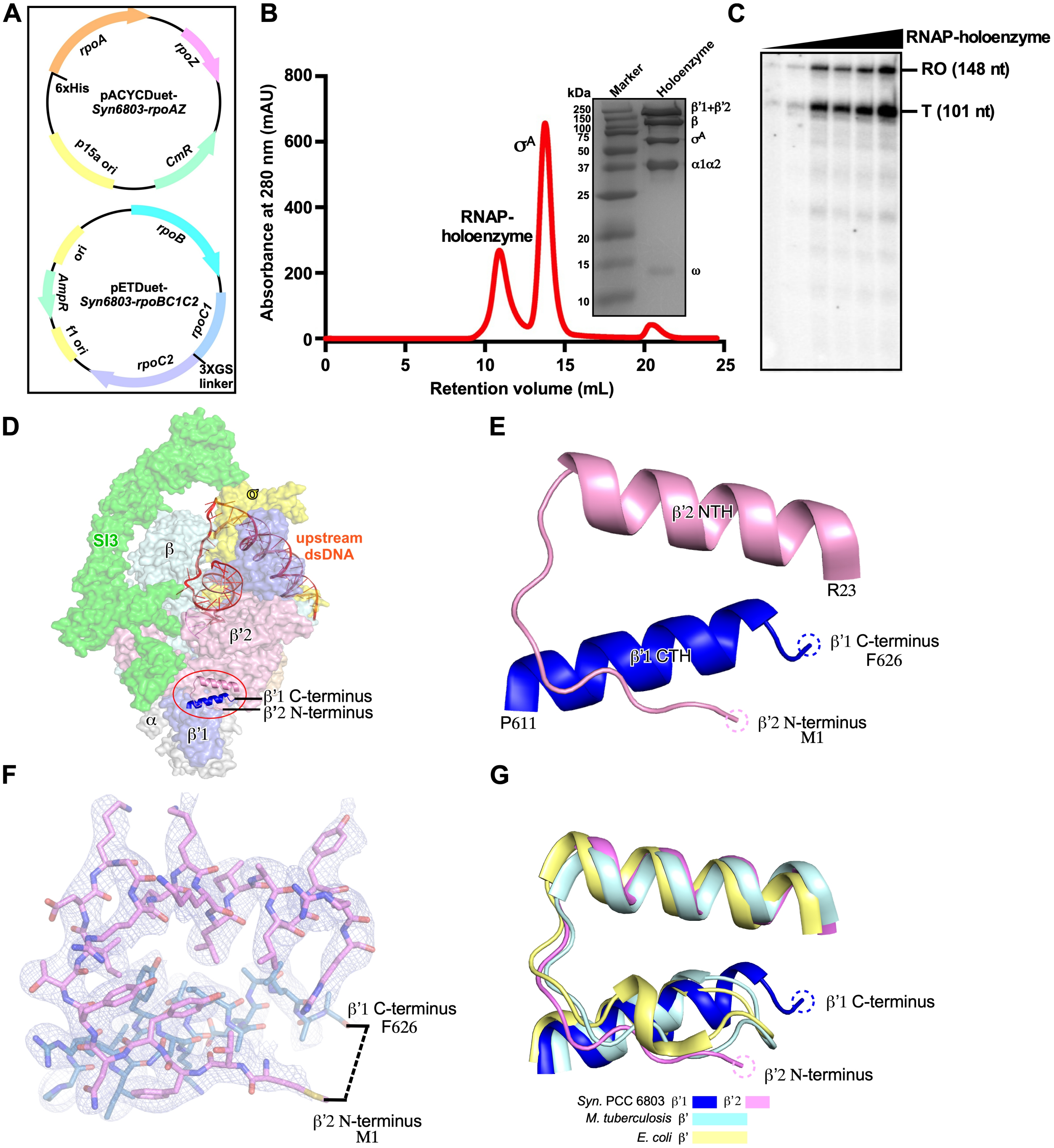

Figure S2. The assembly of *Syn*6803 RNAP-σ^A^ holoenzyme. **(A)** The schematic diagram of constructs used for over-expression of *Syn*6803 RNAP core enzyme in *E. coli*. **(B)** The gel-filtration chromatography result of RNAP-σ^A holoenzyme assembly**,** the SDS-PAGE shows the purity of *Syn*6803 Rpitc. **(C)** The *in vitro* transcription result of *Syn*6803 RNAP-σ^A holoenzyme using N25 promoter. RO, run-off product; T, terminated product. **(D)** The split occurs at the surface of RNAP. **(E)** The split ends remain close to each other. **(F)** The cryo-EM map (mesh) of the split ends. **(G)** structural superimposition of bacterial RNAP suggests that the split ends retain conserved structural fold.

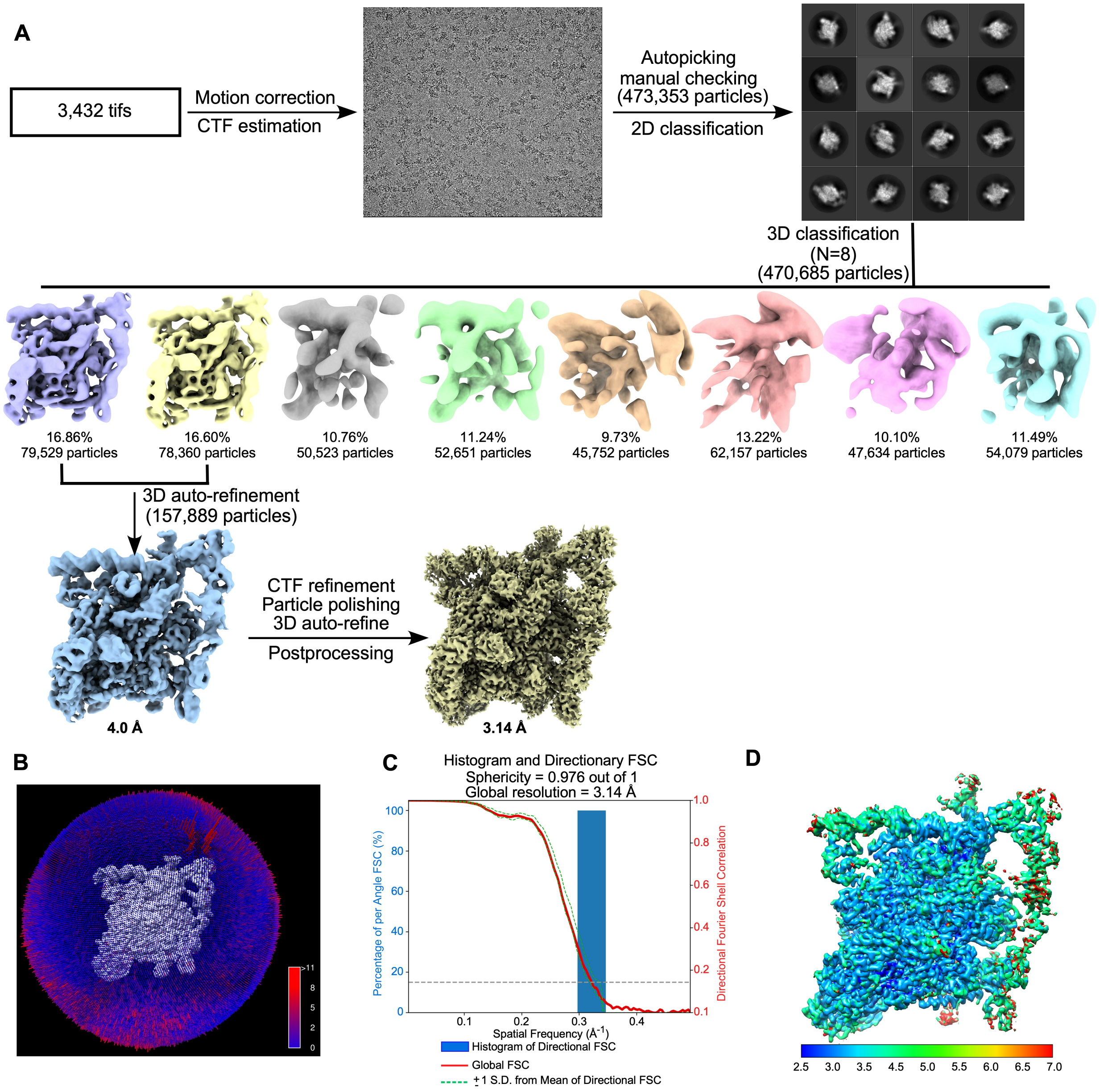

Figure S3. The flow chart of cryo-EM data collection and map calculation for RPitc. (A) The flowchart of data processing. **(B)** The angular distribution of single-particle projections by number of particles of each projection**. (C)** The 3D FSC plot. The dotted line represents 0.143 cutoff of the global FSC curve. **(D)** The cryo-EM map of *Syn*6803 RPitc colored by local resolution.

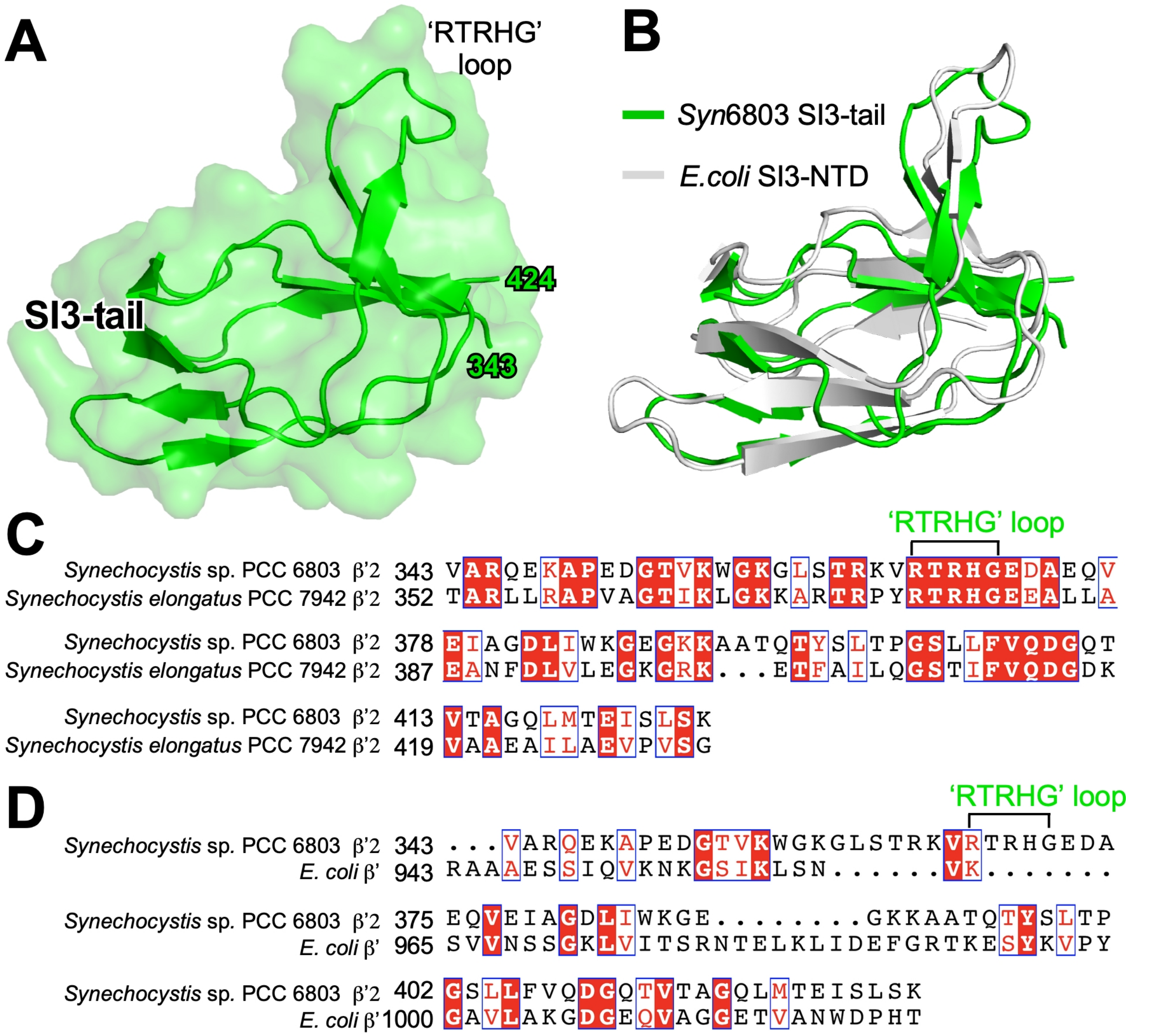

Figure S4. The crystal structure of *Syn*7942 RNAP SI3-tail. (A) The overall crystal structure of *Syn*7942 RNAP SI3-tail. **(B)** The structural superimposition of *Syn*7942 RNAP SI3-tail and *E. coli* SI3-NTD (PDB: 2AUK). **(C)** The protein sequence alignment of SI3-tail of *Syn*6803 and *Syn*7942 RNAP. **(D)** The protein sequence alignment of SI3-tail of *Syn*6803 RNAP and SI3-NTD of *E. coli* RNAP. The 'RTRHG' loop of *Syn*6803 RNAP-SI3 domain is labeled.

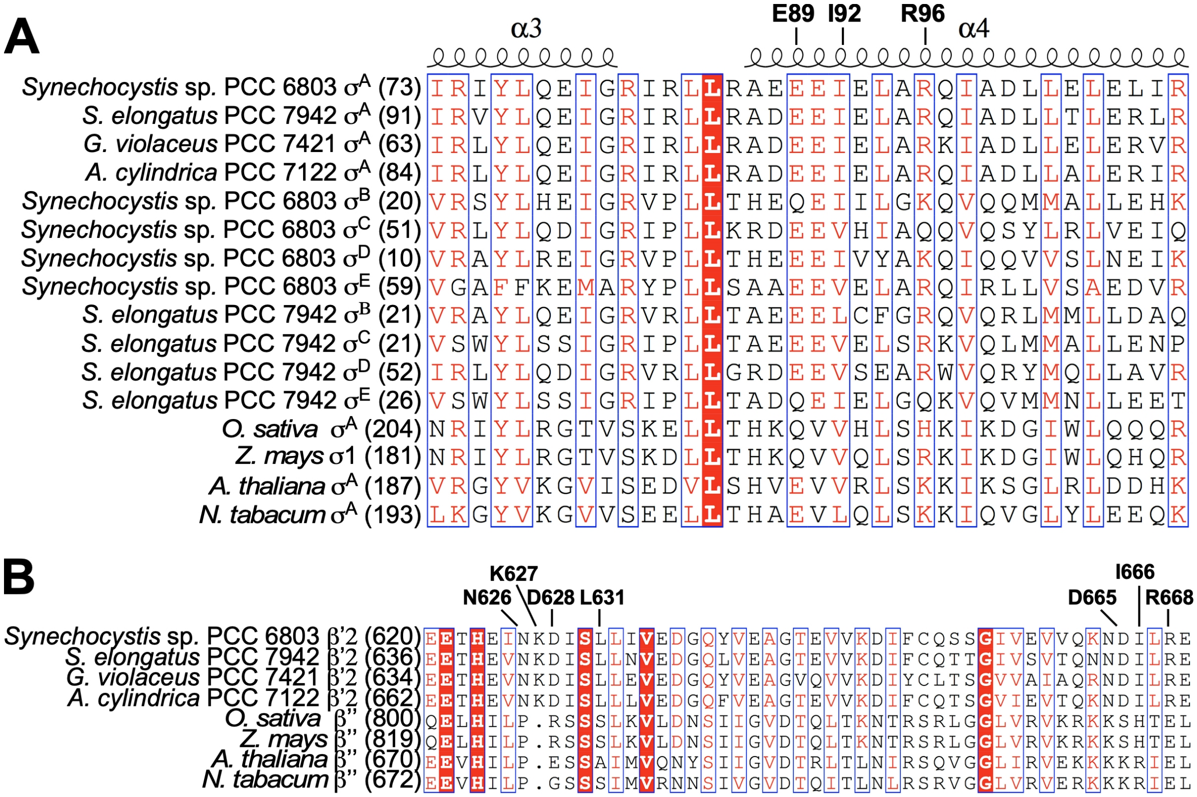

Figure S5. Sequence alignment of SI3-head and σ₂. (A) The protein sequence alignment of the region 2 of σ factors of various cyanobacteria and plant chloroplasts. **(B)** The protein sequence alignment of RNAP SI3-head of various cyanobacteria and plastid-encoded polymerase (PEP) of plant chloroplasts.

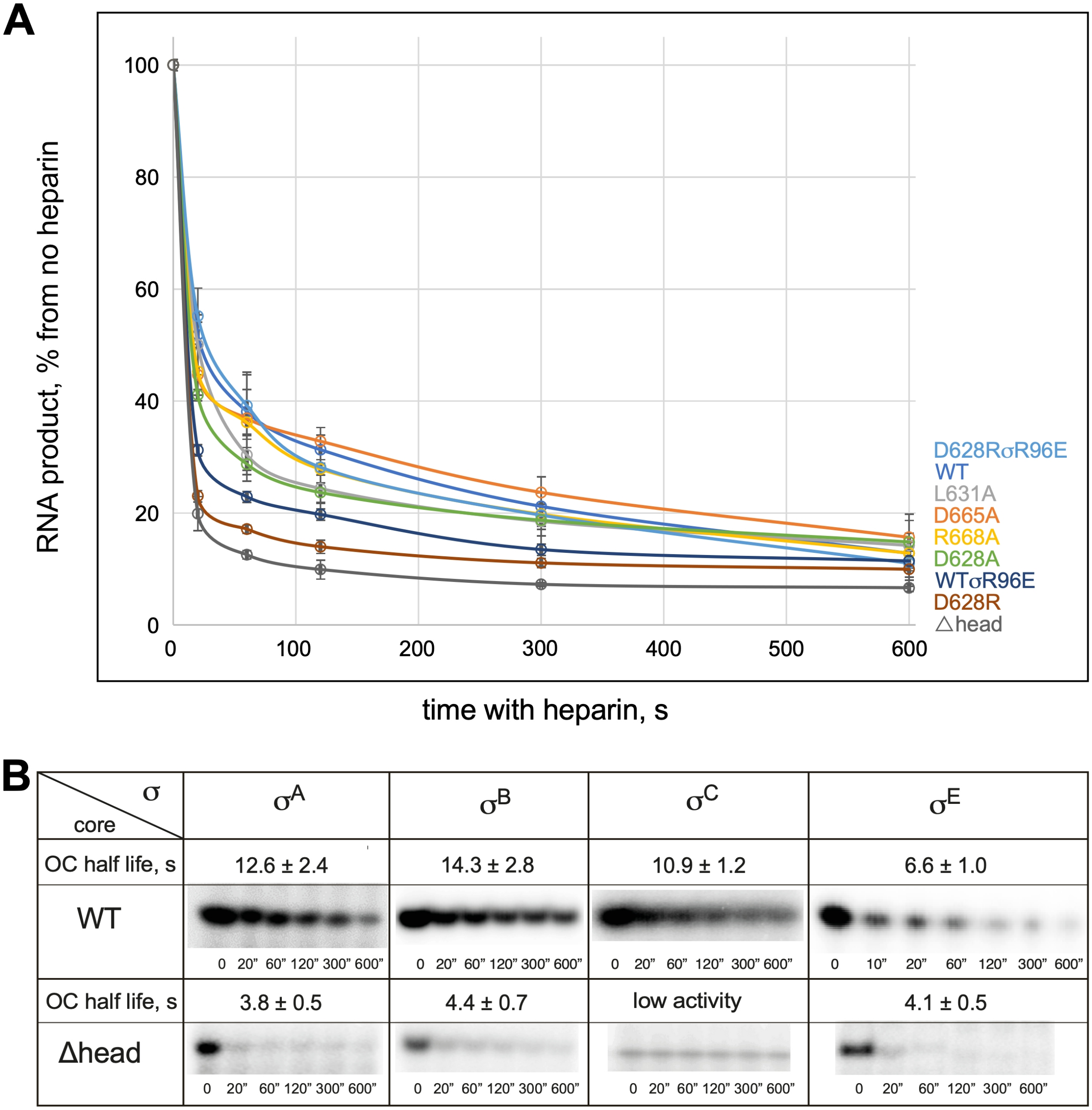

Figure S6. Kinetics of promoter complex decay of WT/mutant *Syn*7942 RNAP holoenzymes in the presence of heparin. (A) The decay cruves of WT/mutant *Syn*7942 RNAP holoenzymes in the presence of heparin. The estimated RPo half-lives is plotted in Fig. 3E. **(B)** Representative gel images and estimated *Syn*7942RPo half-lives (plotted in Fig. 3G) comprising WT/Δhead RNAP and different σ factors from three independent replicates.

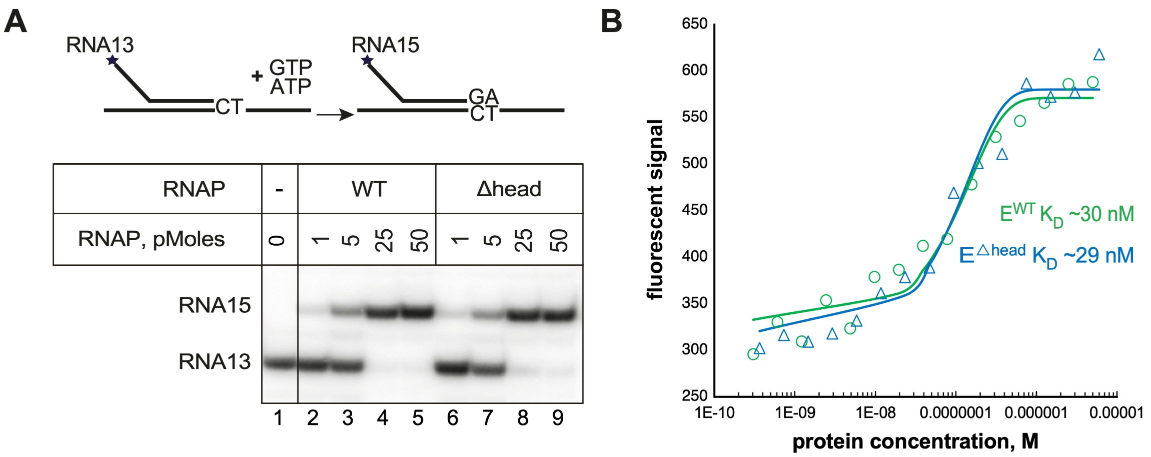

Figure S7. The SI3-head deletion affects neither RNAP catalytic activity nor its affinity to σ. (A) NTP incorporation rate is not affected upon deletion of SI3-head. The 5' end labelled 13 nt long RNA is extended by addition of 100 μM GTP and ATP substrates to 15 nt long RNA using increased concentrations of either WT or $E^{\Delta head}$ RNAP core enzymes. **(B)** Plots of concentration-dependent efficiency of holoenzyme formation and calculated dissociation constants for E^{WT} and $E^{\Delta head}$ holoenzymes measured by microscale thermophoresis.

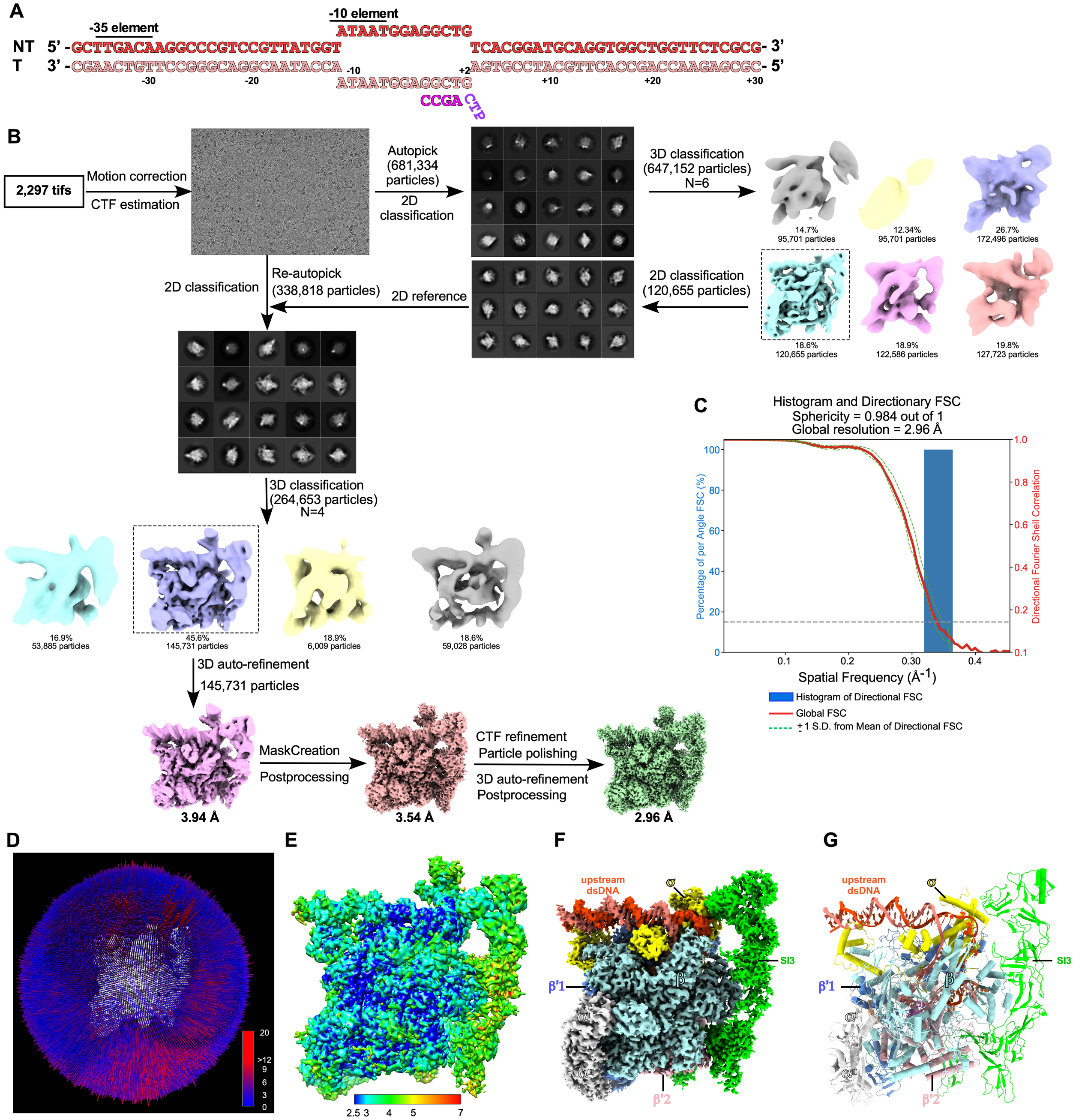

Figure S8. The flow chart of cryo-EM data collection and map calculation for CTP-bound RPitc. (A) The DNA-RNA scaffold used for cryo-EM structure determination. **(B)** The flowchart of data processing. **(C)** The 3D FSC plot. The dotted line represents 0.143 cutoff of the global FSC curve. **(D)** The angular distribution of single-particle projections by number of particles of each projection. **(E)** The cryo-EM maps of *Syn*6803 CTP-bound RPitc colored by local resolution or **(F)** by subunit. **(G)** The structure model of the CTP-bound RPitc.

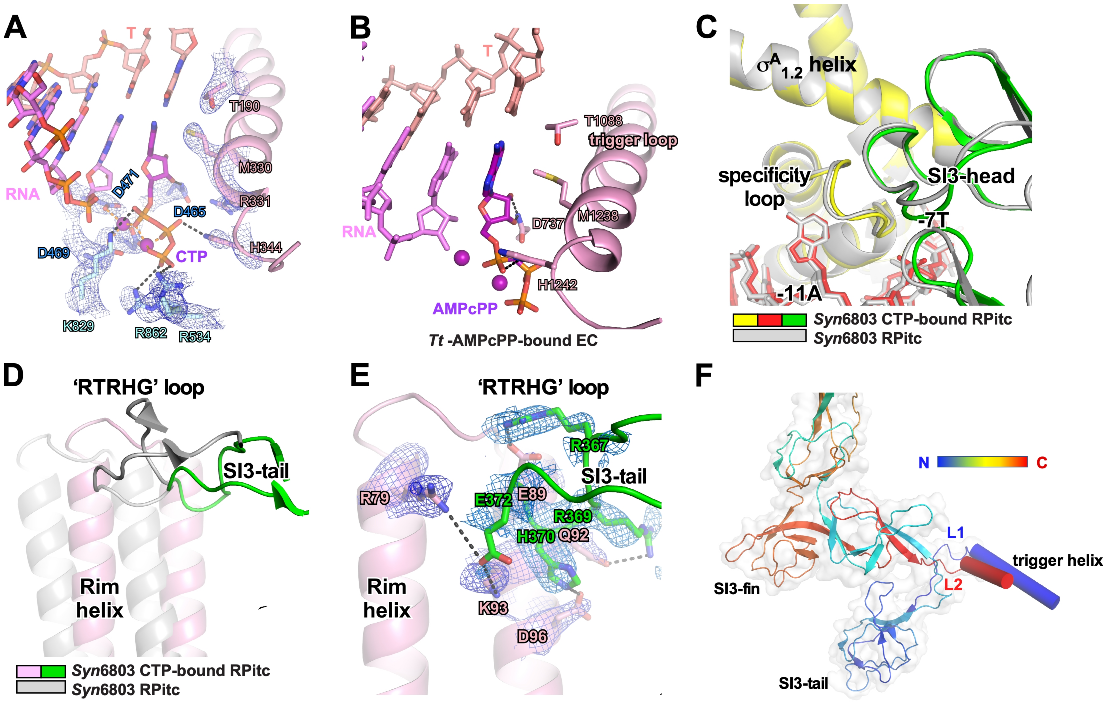

Figure S9. The structural analysis of CTP-bound RPitc. (A) The cryo-EM map and structural model of the detailed interaction between CTP and RNAP residues. **(B)** CMPcPP in the 'i+1' site of *Thermus thermophilus* elongation complex (PDB: 2O5J). **(C)** The structure superimposition between Syn6803 RPitc and CTP-bound RPitc shows that SI3-σ interaction remains intact upon trigger helix refolding. **(D)** The structure superimposition between Syn6803 RPitc and CTP-bound RPitc shows that the rim helices and SI3-tail move together and retain their interaction upon trigger loop folding. **(E)** The cryo-EM map of the SI3-Rim interface in the structure of CTP-bound RPitc. **(F)** The trigger loop refolding indues stretching of the two short linkers, L1 and L2 that connects the trigger helix to SI3-tail and SI3-fin domains, respectively.

Supplemental tables

Table S1. The statistics of *Syn*7942 RNAP SI3-tail crystal structure.

	*S*yn7942 RNAP SI3-tail
**Data collection**	
Space group	P 4_1
Cell dimensions	
a, *b*, *c* (Å)	32.1, 32.1, 82.4
α, β, γ (°)	90.0, 90.0, 90.0
Resolution (Å)	50.00-1.55 (1.58-1.55)
R_{sym} or R_{merge}	0.066 (0.165)
$I/\sigma I$	26.3 (12.3)
Completeness (%)	98.5 (100)
Redundancy	7.2 (6.6)
CC1/2 in highest shell	0.981
**Refinement**	
Resolution (Å)	50.00-1.55
No. reflections	11917
$R_{work/}$ R_{free}	0.198/0.222
No. atoms	
Protein	613
Ligand/ion	0
Water	72
B-factors (Å^2)	
Protein	32.6
Ligand/ion	0
Water	42.5
R.m.s deviations	
Bond lengths (Å)	0.005
Bond angles (º)	0.773

Highest resolution shell is shown in parenthesis.

Table S2. The statistics of *Syn*6803 RPitc cryo-EM structures.

	***Syn*6803 RPitc**	***Syn*6803 CTP-bound RPitc**
**Data collection**		
Number of grids used	1	1
Grid type	C-flat	UltrAuFoil
Microscope/detector	Titan Krios/Gatan K2	Titan Krios/Gatan K3
Voltage (keV)	300	300
Dose rate (e^-/s)	6.7	22.5
Pixel size (Å/pix)	1.014	1.1
Total dose (e^-/ Å^2)	53.6	50
Total exposure time (s)	8	2.69
Number of frames/movie	32	40
Defocus range (µm)	-1.5 to -2.6	-1.2 to -2.2
Number of micrographs	3,432	2,297
Particles used for final map	158,150	145,731
**Model composition**		
Non-hydrogen atoms	29,547	30,339
Protein residues	3,630	3,693
Nucleotide	99	104
Ligands (Zn^{2+}/Mg^{2+}/CTP)	2/1/NA	2/2/1
**Refinement**		
Resolution (Å)	3.14	2.96
Map sharpening B factors [illegible]	64.01	-65.84
Clash score	3.31	3.46
Average B factor (Å^2)		
Protein	20.39	16.61
Nucleotide	39.50	31.58
Ligand	26.99	19.25
RMS deviations		
Bond lengths (Å)	0.003	0.004
Bond angles (°)	0.592	0.762
Ramachandran plot		
Favored (%)	96.28	95.44
Allowed (%)	3.72	4.56
Outliers (%)	0.00	0.00

Table S3. Constructs used in this study.

Constructs	Sources
pET28a-TEV-*Syn*6803-*rpoD*	This study
pET28a-TEV-*Syn*7942-RNAP-SI3-tail	This study
pACYC-*Syn*6803-*rpoAZ*	This study
pCOLA-*Syn*6803-*rpoBC1C2(3x*GS)	This study
pJET-A	This study
pJET-B	This study
pJET-C1	This study
pJET-C2	This study
pJET-Z	This study
pJET-AB	This study
pJET-ABC1	This study
pJET-C2Z	This study
pET28-*Sel7942*-rpoABC1C2Z	This study
pET28-*Sel7942*-rpoD1	This study
pET28-*Sel7942*-rpoD2	This study
pET28-*Sel7942*-rpoD4	This study
pET28-*Sel7942*-rpoD5	This study
pET28-*Sel7942*-rpoD6	This study
pUC19-*Sel7942*-rpoC2/Spec/HR	This study
pUC19-*Sel7942*-rpoC2Δhead/Spec/HR	This study

Table S4. The primers sequences (5' to 3') used in this study.

Oligonucleotides	Sequences
pET28a-*Syn*6803-*rpoD*	F:AAACCTGTATTTTCAGGGCGCCATGGGAATGACCCAGACGAAAGAGCC<br>R:GTGGTGGTGGTGGTGGTGCTCGAGTTAGCGGATATATTCCTTGAG
pET28a-*Syn*7942-RNAP-SI3tail	F:AAACCTGTATTTTCAGGGCGCCATGGCAGGGGAAACTGCCCGTCTGC<br>R:GTGGTGGTGGTGGTGCTCGAGTTAACGACCGCTGACTGGCACC
pACYCDuet-*Syn*6803 *rpoAZ*	F1:CACCATCATCACCACAGCCAGGATCCAATGGCGCAGTTTCAAATTG<br>R1:GACTTAAGCATTATGCGGCCGCTTAAGCTTTAGCCTTTTCG<br>F2:GTATAAGAAGGAGATATACATATGACCAAGCGTAGTAATTTGG<br>R2:GCGGTTTCTTTACCAGACTCGAGTTAATTATCGCTAATAATTTC
pCOLADuet-*Syn*6803-*rpoBC1C2(3x*GS)	F1:CTTTAAGAAGGAGATATACCATGGcaATGACAAACCTTGCCAC<br>R1:GACTTAAGCATTATGCGGCCGCTTACACTTCTTCTTCTTCCA<br>F2:GTATAAGAAGGAGATATACATATGACGTTTTATAACTACAC<br>R2:GGTTTCTTTACCAGACTCGAGTTAATCCTCATCATCCCCAAA<br>F3:AAGTATAAGAAGGAGATATACATAATGAAAGCCCAATCAGAACC<br>R3:GTGTAGTTATAAAACGTCATACTGCCGGAGCCGGAGCCGAACACCAACGCT<br>TCGGCGA
pJET-A	rpoAforward:GTGAGCGGATAACAATTCCCCTCTATCTAGAGATTAAAGAGG<br>AGAAATACTAGATGGTGACATTTCAAGTCGAATG<br>rpoAreverse:ACCTCCACTACTCTAGATCAGGAAACTTTCGATTC<br>pJETAforward:GCGGTAATACGGTTATCCACAGAATCAGGGGATAACGC<br>pJETAreverse:ACCTCCACTACTCTAGATCAGGAAACTTTCGATTC
pJET-B	rpoBforward:GAAAGTTTCCTGATCTAGAGTAGTGGAGGTTACTAGATGGCT<br>GAGCAAACGCAAC<br>rpoBreverse:TTCGGGCTTTGTTAGCAGCCGGATCTTAGTCGTCATCGTCAT<br>CAATG<br>pJETBforward:GAAAGTTTCCTGATCTAGAGTAGTGGAGGTTACTAGATGGC<br>TGAGCAAACGCAAC<br>pJETBreverse:GTGGATAACCGTATTACCGCC
pJET-C1	rpoC1forward:CGATGACGACTAATCTAGAGATTAAAGAGGAGAATACTAGA<br>TGGCGAAGCAGGAACAG<br>rpoC1reverse:CAATTCCCCTCTATCTAGAGATTAAAGAGGAGAATACTAGA<br>TGGCGAAGCAGGAACAG<br>pJETC1forward:CAATTCCCCTCTATCTAGAGATTAAAGAGGAGAATACTAG<br>ATGGCGAAGCAGGAACAG<br>pJETC1reverse:GTGGATAACCGTATTACCGCC
pJET-C2	rpoC2forward:GTTGATCCACTAGTCTAGAGAAAGAGGAGAAATACTAGATG<br>GCAGAAGCGAAAAGC<br>rpoC2reverse:GTGGTGGTGGTGGTGGTCTTCGTCAACATCCAC<br>pJETC2forward:GCGGTAATACGGTTATCCACAGAATCAGGGGATAACGC<br>pJET-C2reverse:GTGGTGGTGGTGGTGGTCTTCGTCAACATCCAC
pJET-Z	rpoZforward:TGTTGACGAAGACCACCACCACCACCACCACTGATCTAGAGA<br>AAGA-GGGGAAATACTAGATGCTCCAGCGCTTCGATC<br>rpoZreverse:TTCGGGCTTTGTTAGCAGCCGGATCTCACCCGCCGATAATCT<br>C<br>pJETZforward:TGTTGACGAAGACCACCACCACCACCACCACTGATCTAGA-<br>GAAAGAGGGGAAATACTAGATGCTCCAGCGCTTCGATC<br>pJETZreverse:GTGGATAACCGTATTACCGCC
pJET-AB	rpoABforward:GCGGTAATACGGTTATCCACAGAATCAGGGGATAACGC<br>rpoABreverse:TTCGGGCTTTGTTAGCAGCCGGATCTTAGTCGTCATCGTCA<br>TCAATG
pJET-ABC1	rpoABC1forward:GCGGTAATACGGTTATCCACAGAATCAGGGGATAACGC<br>rpoABC1reverse:TTCTCCTCTTTCTCTAGACTAGTGGATCAACGCAGTC

pJET-C2Z	rpoC2Zforward:GTTGATCCACTAGTCTAGAGAAAGAGGAGAAATACTAGATGGCAGAAGCGAAAAGC<br>rpoC2Zreverse:GTGGATAACCGTATTACCGCC
pET28-*Sel7942*-rpoABC1C2Z	rpoABC1C2Zforward:GTGAGCGGATAACAATTCCCCTCTATCTAGAGATTAAAGAGGAGAAATACTAGATGGTGACATTTCAAGTCGAATG<br>rpoABC1C2Zreverse:TTCGGGCTTTGTTAGCAGCCGGATCTCACCCGCCGATAATCTC

Supplementary references

1. Z. Otwinowski, W. Minor, Processing of X-ray diffraction data collected in oscillation mode. *Methods Enzymol* **276**, 307-326 (1997).
2. M. Chlenov *et al.*, Structure and function of lineage-specific sequence insertions in the bacterial RNA polymerase beta' subunit. *J Mol Biol* **353**, 138-154 (2005).
3. P. Emsley, K. Cowtan, Coot: model-building tools for molecular graphics. *Acta Crystallogr D Biol Crystallogr* **60**, 2126-2132 (2004).
4. P. D. Adams *et al.*, PHENIX: a comprehensive Python-based system for macromolecular structure solution. *Acta Crystallogr D Biol Crystallogr* **66**, 213-221 (2010).
5. S. Q. Zheng *et al.*, MotionCor2: anisotropic correction of beam-induced motion for improved cryo-electron microscopy. *Nat Methods* **14**, 331-332 (2017).
6. K. Zhang, Gctf: Real-time CTF determination and correction. *J Struct Biol* **193**, 1-12 (2016).
7. R. Fernandez-Leiro, S. H. W. Scheres, A pipeline approach to single-particle processing in RELION. *Acta Crystallogr D Struct Biol* **73**, 496-502 (2017).
8. Y. Zuo, T. A. Steitz, Crystal structures of the E. coli transcription initiation complexes with a complete bubble. *Mol Cell* **58**, 534-540 (2015).
9. S. S. Golden, J. Brusslan, R. Haselkorn, Genetic engineering of the cyanobacterial chromosome. *Methods Enzymol* **153**, 215-231 (1987).
10. A. Riaz-Bradley, K. James, Y. Yuzenkova, High intrinsic hydrolytic activity of cyanobacterial RNA polymerase compensates for the absence of transcription proofreading factors. *Nucleic Acids Res* **48**, 1341-1352 (2020).
